# Control of defensive behavior by the nucleus of Darkschewitsch GABAergic neurons

**DOI:** 10.1093/nsr/nwae082

**Published:** 2024-03-05

**Authors:** Huiying Zhao, Jinrong Liu, Yujin Shao, Xiang Feng, Binhan Zhao, Li Sun, Yijun Liu, Linghui Zeng, Xiao-ming Li, Hongbin Yang, Shumin Duan, Yan-qin Yu

**Affiliations:** Department of Neurology of Second Affiliated Hospital and School of Brain Science and Brain Medicine, Zhejiang University School of Medicine, Hangzhou 310058, China; Nanhu Brain-Computer Interface Institute, Hangzhou 311100, China; Liangzhu Laboratory, MOE Frontier Science Center for Brain Science and Brain-Machine Integration, State Key Laboratory of Brain-Machine Intelligence, Zhejiang University, Hangzhou 311121, China; Department of Neurology of Second Affiliated Hospital and School of Brain Science and Brain Medicine, Zhejiang University School of Medicine, Hangzhou 310058, China; Liangzhu Laboratory, MOE Frontier Science Center for Brain Science and Brain-Machine Integration, State Key Laboratory of Brain-Machine Intelligence, Zhejiang University, Hangzhou 311121, China; Department of Neurology of Second Affiliated Hospital and School of Brain Science and Brain Medicine, Zhejiang University School of Medicine, Hangzhou 310058, China; Liangzhu Laboratory, MOE Frontier Science Center for Brain Science and Brain-Machine Integration, State Key Laboratory of Brain-Machine Intelligence, Zhejiang University, Hangzhou 311121, China; Department of Neurology of Second Affiliated Hospital and School of Brain Science and Brain Medicine, Zhejiang University School of Medicine, Hangzhou 310058, China; Liangzhu Laboratory, MOE Frontier Science Center for Brain Science and Brain-Machine Integration, State Key Laboratory of Brain-Machine Intelligence, Zhejiang University, Hangzhou 311121, China; Department of Neurology of Second Affiliated Hospital and School of Brain Science and Brain Medicine, Zhejiang University School of Medicine, Hangzhou 310058, China; Department of Neurology of Second Affiliated Hospital and School of Brain Science and Brain Medicine, Zhejiang University School of Medicine, Hangzhou 310058, China; Liangzhu Laboratory, MOE Frontier Science Center for Brain Science and Brain-Machine Integration, State Key Laboratory of Brain-Machine Intelligence, Zhejiang University, Hangzhou 311121, China; NHC and CAMS Key Laboratory of Medical Neurobiology, Zhejiang University, Hangzhou 310058, China; Department of Neurology of Second Affiliated Hospital and School of Brain Science and Brain Medicine, Zhejiang University School of Medicine, Hangzhou 310058, China; Liangzhu Laboratory, MOE Frontier Science Center for Brain Science and Brain-Machine Integration, State Key Laboratory of Brain-Machine Intelligence, Zhejiang University, Hangzhou 311121, China; NHC and CAMS Key Laboratory of Medical Neurobiology, Zhejiang University, Hangzhou 310058, China; Key Laboratory of Novel Targets and Drug Study for Neural Repair of Zhejiang Province, School of Medicine, Hangzhou City University, Hangzhou 310015, China; Department of Neurology of Second Affiliated Hospital and School of Brain Science and Brain Medicine, Zhejiang University School of Medicine, Hangzhou 310058, China; Nanhu Brain-Computer Interface Institute, Hangzhou 311100, China; NHC and CAMS Key Laboratory of Medical Neurobiology, Zhejiang University, Hangzhou 310058, China; Department of Neurology of Second Affiliated Hospital and School of Brain Science and Brain Medicine, Zhejiang University School of Medicine, Hangzhou 310058, China; Liangzhu Laboratory, MOE Frontier Science Center for Brain Science and Brain-Machine Integration, State Key Laboratory of Brain-Machine Intelligence, Zhejiang University, Hangzhou 311121, China; NHC and CAMS Key Laboratory of Medical Neurobiology, Zhejiang University, Hangzhou 310058, China; Department of Neurology of Second Affiliated Hospital and School of Brain Science and Brain Medicine, Zhejiang University School of Medicine, Hangzhou 310058, China; Liangzhu Laboratory, MOE Frontier Science Center for Brain Science and Brain-Machine Integration, State Key Laboratory of Brain-Machine Intelligence, Zhejiang University, Hangzhou 311121, China; NHC and CAMS Key Laboratory of Medical Neurobiology, Zhejiang University, Hangzhou 310058, China; Key Laboratory of Novel Targets and Drug Study for Neural Repair of Zhejiang Province, School of Medicine, Hangzhou City University, Hangzhou 310015, China; Department of Neurology of Second Affiliated Hospital and School of Brain Science and Brain Medicine, Zhejiang University School of Medicine, Hangzhou 310058, China; Nanhu Brain-Computer Interface Institute, Hangzhou 311100, China; Liangzhu Laboratory, MOE Frontier Science Center for Brain Science and Brain-Machine Integration, State Key Laboratory of Brain-Machine Intelligence, Zhejiang University, Hangzhou 311121, China; NHC and CAMS Key Laboratory of Medical Neurobiology, Zhejiang University, Hangzhou 310058, China; Key Laboratory of Novel Targets and Drug Study for Neural Repair of Zhejiang Province, School of Medicine, Hangzhou City University, Hangzhou 310015, China

**Keywords:** nucleus of Darkschewitsch, GABAergic population, defensive behavior, freezing, neural circuitry

## Abstract

The nucleus of Darkschewitsch (ND), mainly composed of GABAergic neurons, is widely recognized as a component of the eye-movement controlling system. However, the functional contribution of ND GABAergic neurons (ND_GABA_) in animal behavior is largely unknown. Here, we show that ND_GABA_ neurons were selectively activated by different types of fear stimuli, such as predator odor and foot shock. Optogenetic and chemogenetic manipulations revealed that ND_GABA_ neurons mediate freezing behavior. Moreover, using circuit-based optogenetic and neuroanatomical tracing methods, we identified an excitatory pathway from the lateral periaqueductal gray (lPAG) to the ND that induces freezing by exciting ND inhibitory outputs to the motor-related gigantocellular reticular nucleus, ventral part (GiV). Together, these findings indicate the ND_GABA_ population as a novel hub for controlling defensive response by relaying fearful information from the lPAG to GiV, a mechanism critical for understanding how the freezing behavior is encoded in the mammalian brain.

## INTRODUCTION

Freezing, a defensive response common in rodents and other animals, has been employed as an important index for different fear studies including conditioned fear [1–6] and innate fear [7–11]. Previous studies have identified several brain regions involved in freezing regulation [12]. In particular, the periaqueductal gray (PAG) is a common output for defensive behaviors and is involved in the control of various defensive behaviors [13]. Accumulating evidence suggests that the circuitry that mediates fear responses is complex and involves multiple independent circuits that process different types of fear [12]. However, how the fear response output center selectively generates distinct defensive responses such as freezing, flight or hiding remains largely unknown.

The nucleus of Darkschewitsch (ND), as one of the accessory oculomotor nuclei, was first described in 1889 and was postulated as being involved in eye movement control via zone second cervical spinal cord of the flocculus [14–17]. Immunocytochemical and *in situ* hybridization studies have identified that the majority of neurons in the ND are positive for glutamate decarboxylase (GAD) [18,19]. However, the anatomical outputs and inputs and the physiological functions of GABAergic neurons of the ND remain unclear.

Surprisingly, we observed that the ND_GABA_ neurons were selectively activated by the mouse predator odorant trimethylthiazoline (TMT) and foot shocks. We speculated that the ND_GABA_ neurons might be involved in fear-related-behavior regulation. If so, how the ND_GABA_ populations integrate fear information and control defensive responses remains unknown. To test this hypothesis, we combined electrophysiological recordings, optogenetics, chemogenetics and fiber photometry to investigate the ND_GABA_ neurons and found that ND_GABA_ neurons specifically influenced freezing-like behavioral responses, but not hiding- or fleeing-like responses.

We further identified ND_GABA_ neurons as a downstream target of the lateral PAG (lPAG) that relay fear information to the gigantocellular reticular nucleus, ventral part (GiV) and control the freezing behavior. Lastly, we found that the somatostatin (SOM) but not parvalbumin-positive (PV) GABAergic cell population of ND plays a key role in controlling freezing-like behavior. Together, these findings suggest that the SOM^+^ ND_GABA_ population might be an important behavioral output center that encodes freezing-like defensive behavior.

## RESULTS

### ND_GABA_ neurons selectively respond to fearful stimuli

**Figure 1.**
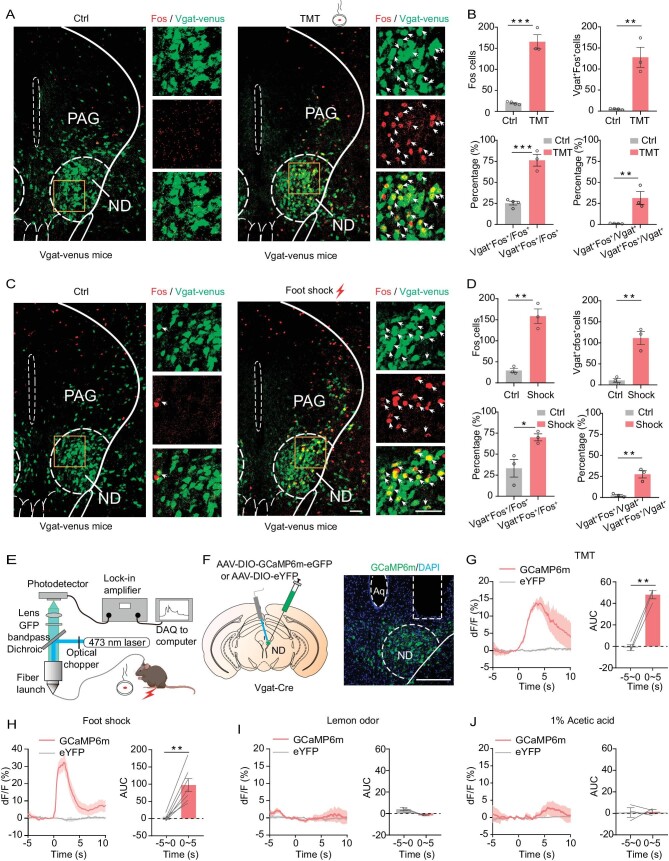
The ND_GABA_ neurons respond to fearful stimuli. (A and C) ND_GABA_ cells and Fos protein-immunopositive cells in response to TMT or control (Ctrl) odor (A) and with or without shock stimulus (C). Scale bar, 50 μm. (B) Top panel, bar graph for number of Fos-positive cells in the ND after TMT or control (Ctrl) odor stimulus (left) and the number of cells of co-expressing Vgat and Fos (right) (Fos_Ctrl_ vs. Fos_TMT_: unpaired t-test, t = 10.24, df = 5, *P* = 0.0002; Vgat^+^Fos^+^_Ctrl_ vs. Vgat^+^Fos^+^_TMT_: unpaired t-test, t = 6.156, df = 5, *P* = 0.0016). Bottom panel, the percentages of Fos^+^ cells expressing Vgat (left, Vgat^+^Fos^+^/Fos^+^_Ctrl_ vs. Vgat^+^Fos^+^/Fos^+^_TMT_: unpaired t-test, t = 7.916, df = 5, *P* = 0.0005) and the percentages of GABAergic (Vgat^+^) cells expressing Fos (right, Vgat^+^Fos^+^/Vgat^+^_Ctrl_ vs. Vgat^+^Fos^+^/Vgat^+^_TMT_: unpaired t-test, t = 4.696, df = 5, *P* = 0.0054). (D) Similar to (B), but for foot shock stimulus (top panel, Fos_Ctrl_ vs. Fos_shocks_: unpaired t-test, t = 7.133, df = 4, *P* = 0.002; Vgat^+^Fos^+^_Ctrl_ vs. Vgat^+^Fos^+^_shocks_: unpaired t-test, t = 6.224, df = 4, *P* = 0.0034. Bottom panel, left, Vgat^+^Fos^+^/Fos^+^_Ctrl_ vs. Vgat^+^Fos^+^/Fos^+^_shocks_: unpaired t-test, t = 3.265, df = 4, *P* = 0.0309; right, Vgat^+^Fos^+^/Vgat^+^_Ctrl_ vs. Vgat^+^Fos^+^//Vgat^+^_shocks_: unpaired t-test, t = 5.566, df = 4, *P* = 0.0051). (E) Schematic of fiber photometry recording. (F) Experimental design for AAV-DIO-GCaMP6m or DIO-eYFP control viral-vector injection (left panel); a sample image of the expression of GCaMP6m in the ND_GABA_ neurons (right panel). Scale bar, 200 μm. (G–J) Left panel, dF/F of GCaMP6m and eYFP signals in response to TMT (G, GCaMP6m, *n* = 3 mice, all trials = 16; eYFP, *n* = 4 mice, all trials = 20), foot shocks (H, GCaMP6m, *n* = 7 mice, all trials = 28; eYFP, *n* = 4 mice, all trials = 16), lemon odor (I, GCaMP6m, *n* = 3 mice, all trials = 15; eYFP, *n* = 4 mice, all trials = 18) and 1% acetic acid (J, GCaMP6m, *n* = 3 mice, all trials = 13; eYFP, *n* = 4 mice, all trials = 17). The shaded areas indicate the SEM; right panel, bar graph of the area under the curve (AUC) for left panel (G, paired t-test, t = 17.33, df = 2, *P* = 0.0033; H, paired t-test, t = 5.653, df = 6, *P* = 0.0013; I, paired t-test, t = 3.299, df = 2, *P* = 0.0809; J, paired t-test, t = 0.1258, df = 2, *P* = 0.9114). *P <  0.05, **P <  0.01, ***P <  0.001. Data represent the mean ± SEM.

Previous studies showed that foot shocks (painful stimulation) and predator-odorant trimethylthiazoline (TMT) successfully provoke a freezing behavior in free-behaving mice [1,3,8,10,11]. To explore the common freezing center in the mouse brain, we first examined Fos protein expression in the whole brain with the existence of freezing induced by foot shocks or TMT. Intriguingly, we observed massive Fos protein expression in the anterior ND (Fig. 1A–D), which has not been linked to fear before. In addition, our *in situ* hybridization studies confirmed that the majority of Fos protein positive cells in the ND were positive for the vesicular GABA transporter (Vgat) (∼70%; Fig. 1A–D), suggesting that GABAergic neurons in the ND might be involved in foot-shock- and TMT-induced fear regulation.

To further examine whether ND_GABA_ neurons specifically respond to different types of fearful stimuli, selectively targeted GABAergic cells of the ND with calcium (Ca^2+^) indicator GCaMP6m by injecting a Cre-dependent adeno-associated virus (AAV) vector carrying the GCaMP6m, and implanted an optical fiber into the ND of Vgat-Cre mice (Fig. 1E and F; Fig. S1A and B). We observed a large increase in Ca^2+^ activity when mice were exposed to TMT (5 s; Fig. 1G) or foot shocks (2 s; Fig. 1H), whereas no significant change in emitted fluorescence signals was observed with control stimulation (i.e. the lemon odor and 1% acetic acid; Fig. 1I and J) or in control-virus-injected mice (Fig. 1G–J).

**Figure 2.**
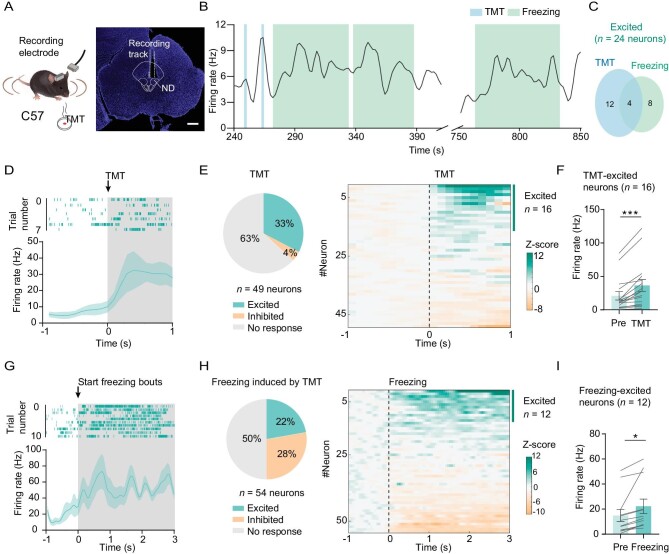
**Figure 2.** ND neurons are excited by predator odor stimulation. (A) Experimental design (left) and coronal section showing the location of stereotrode in the ND (right); scale bar, 500 μm. (B) Sample of spontaneous firing frequency for the ND neuron that was excited when the mouse was sniffing TMT and was in freezing response. (C) Overlap between the proportion of ND neurons that were excited in response to sniffing TMT and freezing response. (D) Sample of spike raster plot (top panel) and spontaneous firing frequency before and during sniffing (shaded) TMT (bottom panel). (E) Left, a pie chart showing the proportion of neurons with a significant increase, a significant decrease or no significant change when sniffing TMT (*n* = 49 neurons from 3 mice); right panel, heatmap of normalized firing rates (Z-score) of ND neurons related to the left panel. (F) The bar graph shows the mean firing frequency before (pre) and during the sniffing of TMT for those TMT-excited neurons (*n* = 16 neurons, paired t-test, t = 4.787, df = 15, *P* = 0.0002). (G) Sample of spike raster plot and spontaneous firing frequency before and during freezing (*n* = 54 neurons). (H) A pie graph (left) and heatmap (right) of neurons with a significant increase, a significant decrease or no significant change during the freezing state (*n* = 54 neurons). (I) The mean firing frequency before (pre) and during freezing for these freezing-excited neurons (*n* = 12 neurons, paired t-test, t = 3.030, df = 11, *P* = 0.0115). *P <  0.05, ***P <  0.001. Data represent the mean ± SEM.

Since fiber photometry does not provide information about the activity of individual cells, to further explore the correlation between individual neuron activity and defensive response, we next performed *in vivo* stereotrode recordings of the ND neurons by implanting a drivable electrode into the anterior ND (Fig. 2A). Although we found some cells were inhibited, the majority of active ND neurons were activated by TMT (33% vs. 4%; Fig. 2B–F). In particular, some of the activated cells dramatically increased firing frequency when mice were in a freezing state (Fig.2B, G–I). Moreover, some of the freeze-responding cells remained active for a long time when the animal showed freezing behavior (Fig. 2B). Together, these findings demonstrate that some ND_GABA_ neurons are highly responsive to fearful stimuli.

### ND_GABA_ neurons control freezing behavior

**Figure 3.**
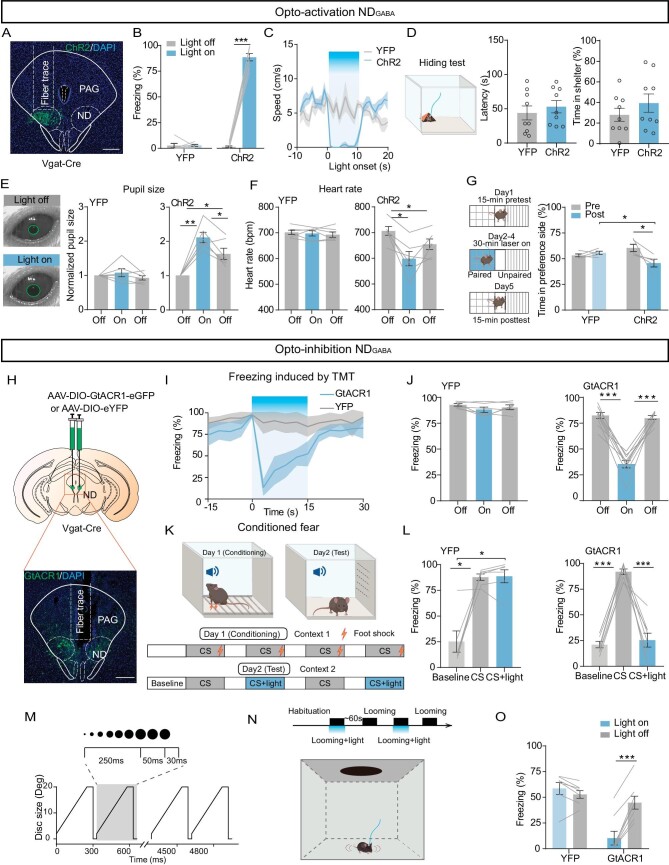
ND_GABA_ neurons are sufficient and necessary to control freezing behavior. (A) A representative image confirming ChR2-eYFP expression in the ND. (B) Percentage of freezing time evoked by optogenetic activation of ND_GABA_ neurons in eYFP-expressing and ChR2-expressing mice (ChR2, *n* = 8 mice, paired t-test, t = 26.67, df = 7, *P* < 0.0001; eYFP, *n* = 6 mice, paired t-test, t = 0.1087, df = 5, *P* = 0.9177). (C) The average speed in open field test during optogenetic activation of ND_GABA_ neurons; blue indicates the light stimulation period (ChR2, *n* = 6 mice; eYFP, *n* = 6 mice). (D) Optogenetic activation of ND_GABA_ neurons had no effect on hiding behavioral response (ChR2, *n* = 9 mice; eYFP, *n* = 9 mice); left, experimental design; middle, latency to nest (unpaired t-test, t = 0.6643, df = 16, *P* = 0.5160) and percentage of hiding time (right, unpaired t-test, t = 1.040, df = 16, *P* = 0.3138). (E) Pupil size in head-fixed mice when 473 nm light stimulated the eYFP-expressing (*n* = 6 mice, one-way repeated-measures ANOVA, F_(1.406, 7.030)_ = 1.280, *P* = 0.3168) or ChR2-expressing ND_GABA_ neurons (*n* = 6 mice, one-way repeated-measures ANOVA, F_(1.957, 9.785)_ = 25.95, *P* = 0.0001). (F) Heart rate measurement of eYFP-expressing (*n* = 6 mice, one-way repeated-measures ANOVA, F_(1.812, 9.061)_ = 0.4136, *P* = 0.6542) or ChR2-expressing animals during optogenetic stimulation of ND_GABA_ neurons (*n* = 6 mice, one-way repeated-measures ANOVA, F_(1.367, 6.834)_ = 11.91, *P* = 0.0084). (G) Left, experimental design for conditional place aversion test; right, percentage of time the eYFP-expressing and ChR2-expressing mice spent in the 473 nm light-stimulation paired compartment (pre: before training; post: after light-stimulation paired training) (two-way repeated-measures ANOVA, F_(1, 8)_ = 7.223, *P* = 0.0276). (H) Experimental design for AAV-DIO-GtACR1 injection (top) and sample image of GtACR1 injected location (bottom). (I) Percentage of freezing time before, during and after 473 nm light was delivered to the ND in eYFP-expressing and GtACR1-expressing mice (eYFP: *n* = 6 mice; GtACR1: *n* = 9 mice). (J) Bar graph from (I) (eYFP, one-way repeated-measures ANOVA, F_(1.501, 7.507)_ = 2.712, *P* = 0.1357; GtACR1, one-way repeated-measures ANOVA, F_(1.818, 14.55)_ = 74.30, *P* < 0.0001). (K) Experimental design of the conditioned fear. (L) Percentage of freezing time when eYFP-expressing or ChR2-expressing mice paired light inhibition of ND_GABA_ neurons in cue-induced freezing tests (eYFP, one-way repeated-measures ANOVA, F_(1.016, 5.082)_ = 18.17, *P* = 0.0076; GtACR1, one-way repeated-measures ANOVA, F_(1.081, 7.564)_ = 67.55, *P* < 0.0001). (M) Expansion of the looming stimulus in time from 2 degrees to 20 degrees for 15 repeated cycles. (N) Timeline for the optogenetic inhibition of the ND during looming stimulus (top) and schematic of the experimental set-up (bottom). (O) Percentage of freezing elicited by looming stimulus combined with or without optogenetic inhibition of the ND_GABA_ neurons (eYFP, *n* = 7 mice; GtACR1, *n* = 8 mice, two-way repeated-measures ANOVA, F_(1, 13)_ = 79.28, *P* < 0.0001). Scale bar, 200 μm. *P <  0.05, **P <  0.01, ***P <  0.001. Data represent the mean ± SEM.

To further investigate the potential causal role of ND_GABA_ neurons in underlying defensive responses, we injected an AAV-carrying Cre-dependent channelrhodopsin-2 (ChR2) into the ND nucleus of Vgat-Cre mice and implanted an optical fiber above the ND (Fig. 3A; Fig. S1C and D). Indeed, in ChR2-expressing mice, when compared to eYFP-expressing mice, optogenetic excitation (20 Hz) of the ND_GABA_ neurons immediately halted their movement (Fig. 3B and C; Video S1). However, activation of ND_GABA_ neurons did not influence other defensive responses, such as hiding behavior (Fig. 3D). Previous studies have shown that fearful stimulation rapidly induces defensive behavior and alters the autonomic nervous system in mice [11,20,21]. The activation of ND_GABA_ neurons indeed resulted in changes in autonomic functions, including a significant increase in pupil size (Fig. 3E) and a decrease in heart rate (Fig. 3F). Conditioning paradigms were used to demonstrate that animals avoid contextual cues previously associated with fear [22–26]. We also observed that optogenetic excitation of ND_GABA_ neurons promotes aversion by using the conditioning paradigm, whereas eYFP-expressing mice spent equal time in both chambers (Fig. 3G). In addition, we repeated this experiment by chemogenetic stimulation of ND_GABA_ neurons (Fig. S2). Compared with the control viral-vector injected animals (AAV-DIO-mCherry), upon clozapine-N-oxide (CNO, 1.0 mg/kg) administration, mice with AAV-DIO-hM3Dq injection showed high-level immobility and anxiety (Fig. S2B–E).

Although our *in situ* hybridization studies confirmed that the majority of neurons in the anterior ND of mice were Vgat positive, many vesicular-glutamate transporter 2 positive cells were observed in the caudal of the ND (Fig. S3A and B). However, activation of glutamatergic neurons in the ND had a much weaker effect on freezing behavior control (Fig. S3C and D).

To investigate the necessity of ND_GABA_ neurons in freezing behavior control, we examined whether optogenetic inactivation of ND_GABA_ neurons by light stimulation (473 nm constant light) of Guillardia theta anion channel rhodopsin 1 (GtACR1) caused a decrease in freezing response. Indeed, inactivation of ND_GABA_ neurons (Fig. 3H and Fig. S4) caused a significant decrease in immobility time induced by TMT (Fig. 3I and J), by an auditory cue in mice with previous fear-conditioned learning (Fig.3K and L), or by looming stimulation (Fig.3M–O), but without affecting the general locomotion in the open field test (Fig. S4B–D) and the hiding behavior provoked by TMT (Fig. S4E–G).

In summary, ND_GABA_ neurons are sufficient and necessary for regulation of freezing behavior.

### Optostimulation of ND_GABA_ neurons did not promote sleep or seizure response

Since the ND has been postulated to be involved in controlling motor behavior, we performed a forced-swimming test with light stimulation of ND_GABA_ neurons and found that light stimulation failed to stop mice attempting to escape from the container, thus the immobility observed above was not a result of a loss of motor ability. However, immobility was observed when placing the animal in a container with less water so that it can stand at the bottom with hind paws (Fig. S5A–C; Video S2). Additionally, a simultaneous electroencephalogram (EEG) recording showed that light-induced brain waves and power intensity are different in rapid-eye-movement (REM) sleep, non-REM sleep and epilepsy [27–31] (Fig. S5D–F). Therefore, the cessation of movement did not result from falling asleep or having a seizure.

### Mapping inputs of ND_GABA_ neurons

**Figure 4.**
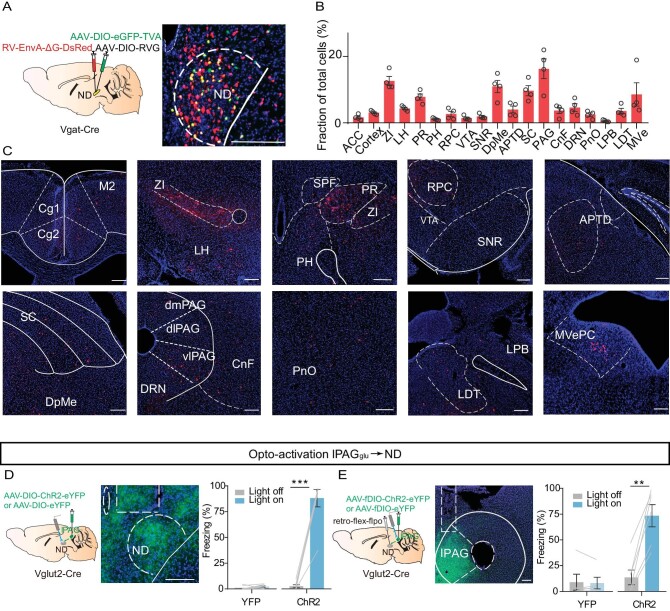
**Figure 4.** The ND as a downstream target of the lPAG controls freezing behavior. (A) Left, schematic showing AAV helper virus (DIO-TVA and DIO-RVG) and RV-EnvA-ΔG-DsRed injections into the ND of Vgat-Cre mice. Right, a coronal brain section showing starter cells in the ND. (B) Quantification of inputs to ND_GABA_ neurons. Data are presented as a percentage of total input neurons counted in each individual brain region (*n* = 4 mice). (C) Sample images showing DsRed cells in different brain regions. ACC, anterior cingulate cortex; ZI, zona incerta; LH, lateral hypothalamus; PR, prerubral field; PH, posterior hypothalamic area; RPC, red nucleus, parvicellular part; VTA, ventral tegmental area; SNR, substantia nigra, reticular part; DpMe, deep mesencephalic nucleus; APTD, anterior pretectal nucleus, dorsal part; SC, superior colliculus; PAG, periaqueductal gray; CnF, cuneiform nucleus; DRN, dorsal raphe nucleus; PnO, pontine reticular nucleus, oral part; LPB, lateral parabrachial nucleus; LDT, laterodorsal tegmental nucleus; MVe, medial vestibular nucleus. (D) Optogenetic activation of the lPAG_Glu_ projections in the ND. Left, experimental design; middle, optical fiber implanted location; right, percentage of freezing evoked by optogenetic excitation of lPAG_Glu_ projections in the ND (eYFP, *n* = 7 mice, paired t-test, t = 1.015, df = 6, *P* = 0.3492; ChR2, *n* = 7 mice, paired t-test, t = 10.59, df = 6, *P* < 0.0001). (E) Left, experimental design for selective manipulation of ND-projecting lPAG cells by using a retrograde tracing strategy; middle, a sample image showing AAV-ChR2 expression in the lPAG; right, percentage of freezing evoked by light stimulation of the ND-projecting lPAG_Glu_ neurons (eYFP, *n* = 5 mice, paired t-test, t = 0.3573, df = 4, *P* = 0.7390; ChR2, *n* = 6 mice, paired t-test, t = 6.709, df = 5, *P* = 0.0011). Scale bar, 200 μm. **P <  0.01, ***P <  0.001. Data represent mean ± SEM.

To identify how ND_GABA_ neurons integrate into the brain's ‘encephalic aversion system’ [32] to support freezing behavior, we used a rabies-virus-based (RV) trans-synaptic tracing strategy [33] to map whole-brain monosynaptic inputs onto ND_GABA_ neurons (Fig. 4A–C). Histological analysis verified the localization of starter cells in the ND (Fig. 4A). We observed the vast majority of RV-labeled cells located in fear-related brain regions such as the zona incerta (ZI) [34,35], the lateral hypothalamus (LH) [36,37], the superior colliculus (SC) [38–40], the lateral parabrachial nucleus (LPB) [41,42] and the lPAG [43] (Fig. 4B and C).

The PAG is an essential common output center for defensive behavior, as demonstrated before [12,13,44]. In particular, the glutamatergic population of the ventrolateral periaqueductal gray (vlPAG_Glu_) is involved in freezing-like behavior control [45–48]. So, we hypothesized that the ND_GABA_ neurons might receive lPAG inputs and encode freezing-like defensive behavior. To investigate whether the lPAG_Glu_→ND pathway is involved in any defensive behavior control, we optogenetically activated lPAG glutamatergic terminals in the ND and found that photostimulation immediately induced a freezing response (Fig. 4D). Additionally, to exclude the influence of the ND passing fibers from the PAG to other brain regions evoke the freezing behavior, we used dual-switch AAV-borne molecular payload technology [49] to express ChR2 genetically in lPAG_Glu_ neurons that specifically project to the ND. As expected, excitation of ND-projecting lPAG_Glu_ neurons promoted freezing behavior as well, whereas the control viral-vector injected animals showed no effects (Fig. 4E). These results verified that the ND, as a downstream target of lPAG_Glu_ neurons, controls freezing behavior.

### ND_GABA_ neurons relay threat information from the PAG to the medulla

**Figure 5.**
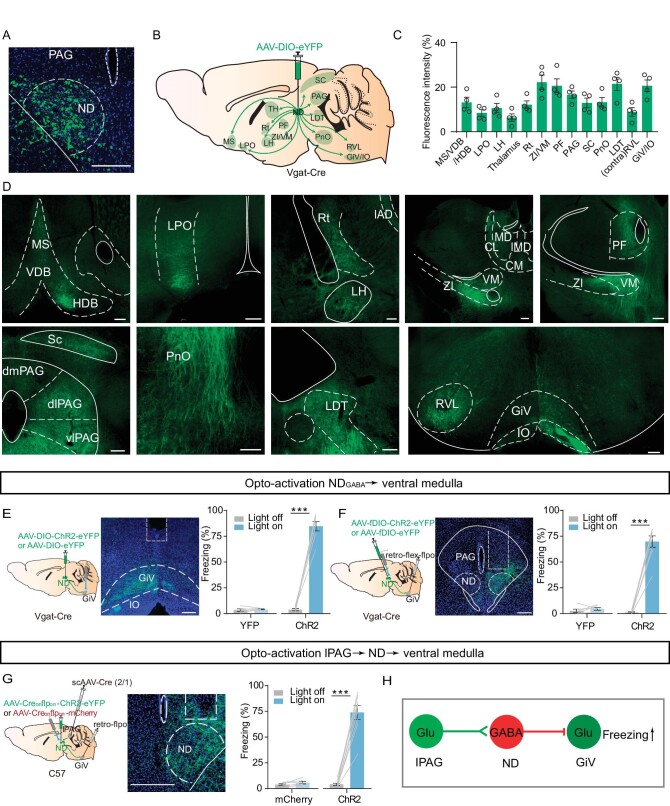
ND_GABA_ neurons relay fearful information from the PAG to the medulla. (A) A coronal brain section showing eYFP-expressing ND_GABA_ neurons in the ND. To map ND_GABA_ projection, AAV-DIO-eYFP was injected into the ND of Vgat-Cre mice. (B) Schematic for the projections of ND_GABA_ neurons. (C) Mean eYFP fluorescence intensity in different brain areas (*n* = 4 mice). (D) Sample images of eYFP-expressing projections to different brain regions from the ND. MS, medial septal nucleus; VDB, nucleus of the vertical limb of the diagonal band; HDB, nucleus of the horizontal limb of the diagonal band; LPO, lateral preoptic area; Rt, reticular nucleus; VM, ventromedial thalamic nucleus; PF, parafascicular thalamic nucleus; RVL, rostroventrolateral reticular nucleus; GiV, gigantocellular reticular nucleus, ventral part; IO, inferior olive. (E) Light stimulation of ND_GABA_ projections to the GiV. Left, experimental design; middle, a sample of optical fiber implanted site; right, percentage of freezing evoked by light stimulation (eYFP, *n* = 5 mice, paired t-test, t = 0.6637, df = 4, *P* = 0.5432; ChR2, *n* = 7 mice, paired t-test, t = 15.45, df = 6, *P* < 0.0001). (F) Optogenetic activation of GiV-projecting ND_GABA_ neurons. Left, experimental design; middle, a sample image of virus expressing; right, percentage of freezing evoked by light-stimulation (eYFP, *n* = 6 mice, paired t-test, t = 0.9611, df = 5, *P* = 0.3807; ChR2, *n* = 8 mice, paired t-test, t = 12.96, df = 7, *P* < 0.0001). (G) Left, experimental design for optogenetic activation of the GiV-projecting and lPAG-targeted ND neurons; middle, a sample image of ChR2-expressing cells in the ND; right, the percentage of freezing (mCherry, *n* = 6 mice, paired t-test, t = 1.715, df = 5, *P* = 0.1471; ChR2, *n* = 8 mice, paired t-test, t = 11.19, df = 7, *P* < 0.0001). (H) Working model of the lPAG_Glu_-ND_GABA_-GiV_glu_ pathway. Scale bar, 200 μm. *P <  0.05, **P <  0.01, ***P <  0.001. Data represent the mean ± SEM.

Next, to investigate the downstream target of ND_GABA_ neurons underlying immobility, we injected anterograde AAV-DIO-eYFP into the ND of Vgat-Cre mice and examined the anatomical organization of the ND GABAergic projection by brain-wide imaging of the ND neuronal axons expressing eYFP. We observed that GABAergic neurons project primarily to brain regions involved in movement control, such as the SC and GiV, and also project to several regions involved in fear regulation, including the laterodorsal tegmental nucleus (LDT), the PAG, the LH and the ZI (Fig. 5A–D). Given that previous studies have shown that the GiV is involved in immobility control [46,50], we speculated that the GiV as the downstream of ND_GABA_ neurons controls freezing-like behavior. We then examined the effects of optogenetically exciting ND_GABA_ neuronal terminals in the GiV. Compared to eYFP-expressing mice, ChR2-expressing mice showed immediately halted movement (Fig. 5E), an effect similar to that of direct stimulation of GABAergic neurons in the ND. To exclude the possibility of backpropagation, we also selectively manipulated the GiV-projecting GABAergic neurons in the ND and observed a robust freezing-like response (Fig. 5F). To further test whether the GiV is one of the downstream targets of ND_GABA_ neurons in freezing control, we directly manipulated GABAergic and glutamatergic neurons in the GiV and found that optogenetic inhibition of GiV_Glu_ neurons but not GiV_GABA_ neurons reliably promoted the freezing response (Fig. S6). More importantly, when selectively manipulating the GiV projecting GABAergic neurons in the ND, which receive lPAG inputs, we observed a significant freezing-like response as well (Fig. 5G).

In summary, we identified ND_GABA_ neurons, as a downstream target of the lPAG, relaying threatening information to the GiV to produce freezing behavior (Fig. 5H).

### Somatostatin GABAergic neurons in the ND control freezing-like behavior

**Figure 6.**
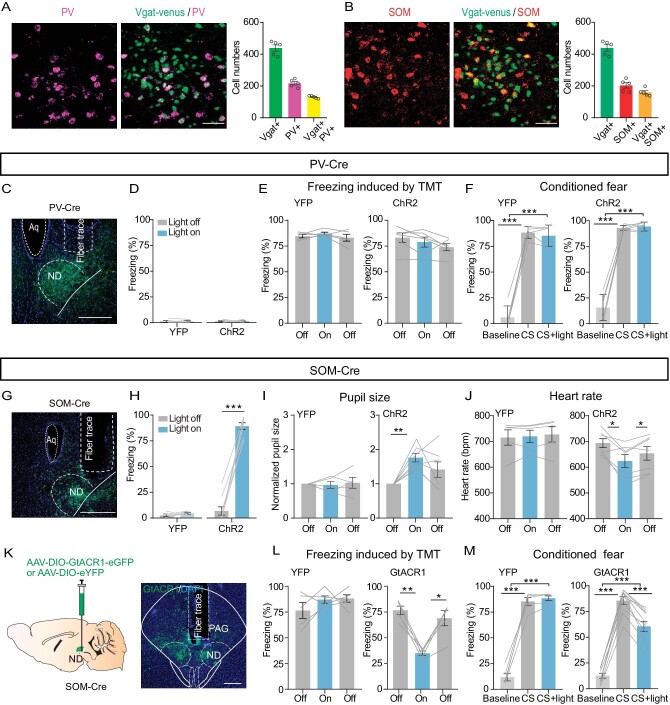
Somatostatin (SOM) GABAergic neurons in the ND control freezing behavior. (A) Left panel, a sample image of the immunostaining of PV in the ND of Vgat-venus mice (scale bar, 50 μm); right, cell count of Vgat^+^, PV^+^ and Vgat^+^PV^+^ in the ND (*n* = 5 mice). (B) Similar to (A) but for SOM immunostaining in the ND (scale bar, 50 μm; *n* = 5 mice). (C) A sample image of PV-ChR2-expressing in the ND. Scale bar, 200 μm. (D) Percentage of freezing time evoked by light stimulation of PV neurons in the ND of eYFP-expressing or ChR2-expressing mice (eYFP, *n* = 5 mice, paired t-test, t = 0.1963, df = 4, *P* = 0.8540; ChR2, *n* = 7 mice, paired t-test, t = 0.3318, df = 6, *P* = 0.7513). (E, F) The effects of optogenetic activation of PV neurons in the ND on TMT (eYFP, *n* = 5 mice, one-way repeated-measures ANOVA, F_(1.709, 6.836)_ = 1.219, *P* = 0.34347; ChR2, *n* = 6 mice, one-way repeated-measures ANOVA, F_(1.646, 8.230)_ = 3.365, *P* = 0.0910) or conditioned cue-induced freezing (F, eYFP, *n* = 5 mice, one-way repeated-measures ANOVA, F_(1.561, 6.245)_ = 201.7, *P* < 0.0001; ChR2, *n* = 6 mice, one-way repeated-measures ANOVA, F_(1.063, 5.314)_ = 192.5, *P* < 0.0001). (G) A sample image of ChR2-expressing in the ND of SOM-Cre mice, scale bar, 200 μm. (H) The percentage of freezing evoked by optogenetic stimulation of SOM neurons in eYFP- or ChR2-expressing mice (eYFP, *n* = 5 mice, paired t-test, t = 0.9671, df = 4, *P* = 0.3883; ChR2, *n* = 9 mice, paired t-test, t = 15.49, df = 8, *P* < 0.0001). (I) Pupil size (eYFP, *n* = 5 mice, one-way repeated-measures ANOVA, F_(1.572, 6.287)_ = 0.1149, *P* = 0.8488; ChR2, *n* = 7 mice, one-way repeated-measures ANOVA, F_(1.466, 8.793)_ = 7.372, *P* = 0.0176). (J) Heart rate (eYFP, *n* = 5 mice, one-way repeated-measures ANOVA, F_(1.112, 4.447)_ = 0.9374, *P* = 0.3948; ChR2, *n* = 7 mice, one-way repeated-measures ANOVA, F_(1.214, 7.283)_ = 10.30, *P* = 0.0119). (K) Optogenetic inhibition of SOM neurons in the ND. Left, experimental design; right, a sample image of GtACR1 expression in the ND. Scale bar, 200 μm. (L, M) The effects of optogenetic inhibition of SOM neurons in the ND on TMT (eYFP, *n* = 5 mice, one-way repeated-measures ANOVA, F_(1.730, 6.922)_ = 1.134, *P* = 0.3652; GtACR1, *n* = 5 mice, one-way repeated-measures ANOVA, F_(1.683, 6.734)_ = 24.52, *P* = 0.0010) or conditioned cue-induced freezing (M, eYFP, *n* = 5 mice, one-way repeated-measures ANOVA, F_(1.205, 4.819)_ = 209.4, *P* < 0.0001; GtACR1, *n* = 10 mice, one-way repeated-measures ANOVA, F_(1.972, 17.75)_ = 24.52, *P* < 0.0001). *P <  0.05, **P < 0.01, ***P <  0.001. Data represent the mean ± SEM.

Fluorescence *in situ* hybridization (FISH) showed that most venus-labeled ND_GABA_ neurons in the Vgat-venus mice expressed SOM or PV (Fig. 6A and B; Vgat + SOM+: ∼37%; Vgat + PV+: ∼30%) and few cells with SOM and PV co-labeling (∼4.5%), suggesting the presence of at least two subtypes of GABAergic cells. Next, to test the role of PV and SOM neurons in freezing control, we injected a Cre-dependent AAV-vector carrying the ChR2 or GtACR1 into the ND of PV-Cre or SOM-Cre mice (Fig. 6C, G and K). Optogenetic excitation with ChR2 of the PV neurons neither enhanced freezing behavior (Fig. 6D) nor reduced freezing induced by TMT or conditioning cue with previous fear-conditioned learning (Fig. 6E and F). However, activation of the SOM neurons caused a strong increase in freezing response (Fig. 6G and H), pupil size (Fig. 6I) and a decrease in heart rate (Fig. 6J). Moreover, light inhibition of SOM neurons in the ND strongly decreased the freezing response induced by TMT or conditioning cue in mice with previous fear-conditioned learning (Fig. 6K–M). Whereas, in eYFP-expressing mice without ChR2 or GtACR1, light stimulation had no such effects. Together, these results indicate that SOM neurons in the ND are particularly important for defensive behavior control.

## DISCUSSION

One of the major goals of neuroscience research is to understand how fear results in the expression of a range of adaptive or defensive behaviors which have a profound role in the survival of a species in a constantly changing environment. Indeed, it has long been assumed that an ‘encephalic aversion system’ [32] exists in the mammalian brain and that this system, in the presence of a threat, generates defensive responses such as freezing, fleeing or hiding [21]. However, fear is an incredibly complex emotion, and although a brain structure called the amygdala is often considered the ‘fear center’ in the brain, it is certain that the ability of the brain to generate and control defensive reactions involves many different brain regions [12]. Although different types of threats are detected by the brain via different sensory modalities, we believe that there are common threat integration and fear response output centers in the brain [12,13,51,52]. The PAG, as a common output for defensive behaviors [13,53]. In particular, the glutamatergic outputs to pre-motor targets in the medulla play a critical role in encoding freezing-like behavior [46] and the lPAG glutamatergic neurons mainly innervate medulla GABAergic neurons [54]. This suggests that lPAG glutamatergic neurons promote immobility by directly activating medullary GABAergic neurons. We unexpectedly found that the ND, as a downstream target of the lPAG, specifically mediates freezing-like defensive behavior through its inhibitory projections to the GiV (a subregion of the medulla), in part at least. We discovered that selective activation of ND_GABA_ neurons is sufficient to induce freezing-like behavior evaluated by previous studies [55,56]: (i) defensive behaviors such as freezing [21]; (ii) altering autonomic function such as changing heart rate during fear and anxiety [11]; (iii) pupil size, which is greatly increased during fear and aggression [57,58], but is strongly reduced during drowsiness and sleep [59,60]; and (iv) EEG (electroencephalogram) and EMG (electromyography)recording [11,27,28,31]. Moreover, the ND_GABA_ neurons have connections with many fearful-behavior-related brain regions (Figs 4 and 5; i.e. the PAG).

lPAG glutamatergic outputs can directly regulate the medulla to encode freezing-like defensive behavior. Here, we identified that medulla-projecting ND_GABA_ neurons, as a downstream target of lPAG glutamatergic neurons, encode freezing-like defensive behavior as well. This supports the existence of multiple parallel neuronal circuits in the brain to control a defensive response in order to facilitate more flexible switching between different defensive behaviors.

Medullary glutamatergic neurons are essential to support high-speed locomotion [61]. Thus, we hypothesized that ND_GABA_ neurons encode immobility, at least in part, by inhibiting medullary glutamatergic neurons, and result in halted locomotion. In addition, the GiV directly sends projections to the spinal cord and modulates cardiac response, thus the autonomic response evoked by manipulating the ND_GABA_ neurons could also result from the medulla [50,62–64]. We cannot exclude the possibility that other resources [65] in the medulla mediate ND_GABA_ projections and evoke defensive behavior and autonomic responses.

The GABAergic population in the ND contains both SOM^+^ and PV^+^ GABAergic neurons (Fig. 6), at least. The freezing-promoting effect is partly attributable to the SOM^+^ GABAergic neurons (Fig. 6G–M). Although previous studies have shown the reciprocal connections between SOM^+^ and PV^+^ neurons within the cortex or brainstem nucleus and their opposite roles in modulating conditioned social fear or innate fear [11,66], activation of PV^+^ GABAergic neurons in the ND had no effect on freezing regulation (Fig. 6C–F). However, we cannot exclude the possibility that the two populations have reciprocal connections in the ND, and they have distinct roles in controlling different defensive responses; it will be an important future research topic.

Fear is highly correlated with anxiety, panic disorder and post-traumatic stress disorder. However, there are currently no effective clinical interventions or treatments for these disorders. In the study, we identified an inhibitory subtype of ND neurons as a novel hub in the control of freezing behavior. Our results advance the current understanding of how threats selectively trigger freezing, a specific defensive response, via lPAG_Glu_-ND_GABA_-GiV_Glu_ circuitry, and provide precise anatomical and functional information that is important for the discovery and development of new therapeutic interventions for mood disorders.

## MATERIALS AND METHODS

### Mouse line

Adult C57BL/6J, Vgat-IRES-Cre (Jackson, strain name *Slc32a1^tm2(cre)Lowl^/J*, stock number 016962), Vglut2-IRES-Cre (Jackson, strain name *B6J;129S6-(FVB)-Slc17a6^tm2(cre)LowI^/MwarJ*, stock number 028863), SOM-Cre (Jackson, strain name *Sst^tm2.1(cre)Zjh^/J*, stock number 013044), PV-Cre (Jackson, strain name *B6.129P2-Pvalb^tm1(cre)Arbr^/J*, stock number 017320) and Vgat-Venus mice were used for the experiments. C57BL/6J mice were purchased from Vital River Experiment Animal Co., Ltd. Mice were housed 3–5 mice per cage on a 12 h light/dark cycle with lights on from 7 : 00 a.m. to 7 : 00 p.m. Mice had free access to food and water *ad libitum*. The mice were maintained at the animal facility of Zhejiang University, and all animal procedures were approved by the Institutional Animal Care and Use Committee (IACUC).

Detailed materials and methods are available in the supplementary data.

## Supplementary Material

nwae082_Supplemental_File

## Data Availability

All custom code used for analysis in this manuscript is available on request. All data are reported in the main text and supplementary materials, stored at Zhejiang University and available upon request.
