# Supplementary material for: Control of defensive behavior by the nucleus of Darkschewitsch GABAergic neurons
Source: Natl Sci Rev. 2024 Mar 5;11(4):nwae082. doi: 10.1093/nsr/nwae082 (PMC11057443; doi:10.1093/nsr/nwae082)
Supplement: nwae082_Supplemental_File [file nwae082_supplemental_file.zip › Supplementary Data.docx]

**Fig. S1**


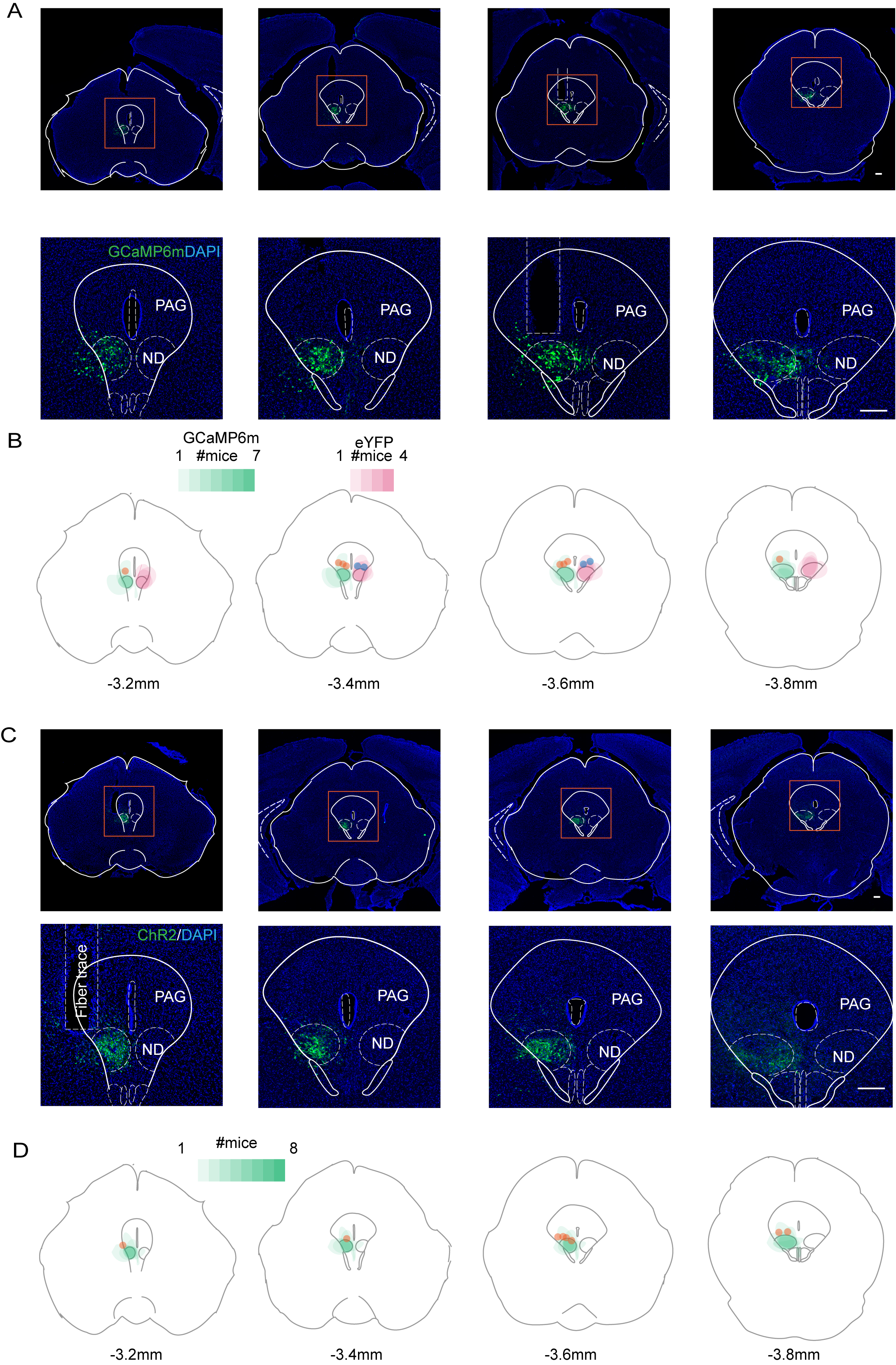


**Fig. S1 GCaMP6m and ChR2 expression in the ND of Vgat-Cre mice**

(A) Coronal brain section samples show viral injection sites (Blue, DAPI; green, GCaMP6m).

(B) Serial reconstruction of fiber implantation and viral injection sites in the ND (top; green/pink color code represents individual mice; yellow/blue dots represent the implanted sites).

(C) Coronal brain section samples show viral injection sites (Blue, DAPI; green, ChR2-eYFP).

(D) Serial reconstruction of fiber implantation and viral injection sites in the ND (top; green color code represents individual mice; yellow dots represent the implanted sites).

Scale bar, 200 μm.

**Fig. S2**


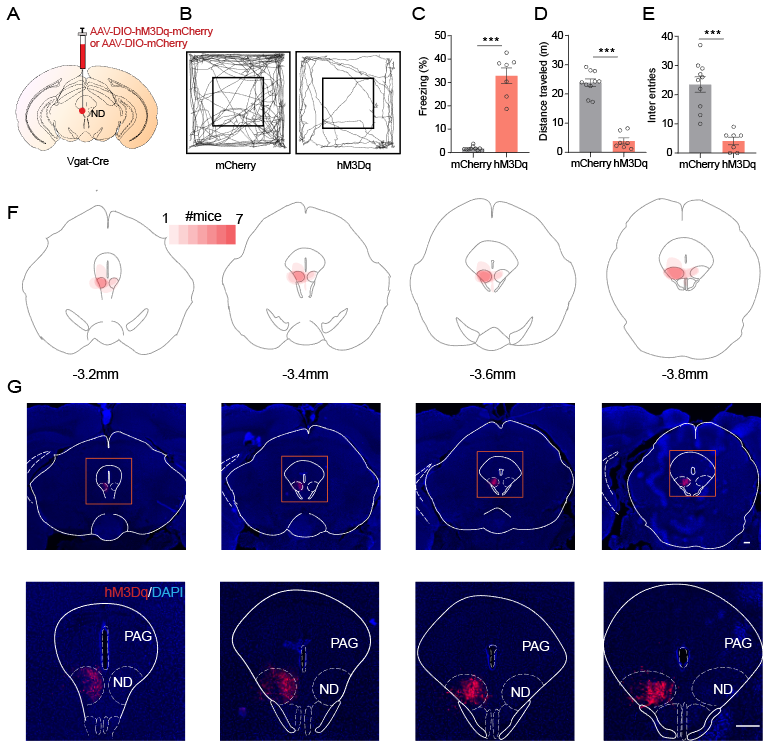


**Fig. S2 Chemogenetic activation of GABAergic neurons in the ND promoted immobility and anxiety**

(A) Schematic for hM3Dq viral injection.

(B) Representative trajectories of hM3Dq-expressing or mCherry-expressing mice during open field test after CNO administration (i.p., 1mg/kg).

(C) Percentage of freezing (mCherry: n=10 mice; hM3Dq, n= 7 mice, unpaired t-test, t=11.23, df=15, p<0.0001).

(D) Traveled distance (mCherry: n=10 mice; hM3Dq, n= 7 mice, unpaired t-test, t=11.33, df=15, p<0.0001).

(E) Center entered times (mCherry: n=10 mice; hM3Dq, n= 7 mice, unpaired t-test, t=5.753, df=15, p<0.0001).

(F) Serial reconstruction of viral injection sites in the ND.

(G) Coronal brain section samples show viral injection sites.

Scale bar, 200 μm. **p*< 0.05, ***p*< 0.01, ****p*< 0.001. Data represent the mean ± SEM.

**Fig. S3**


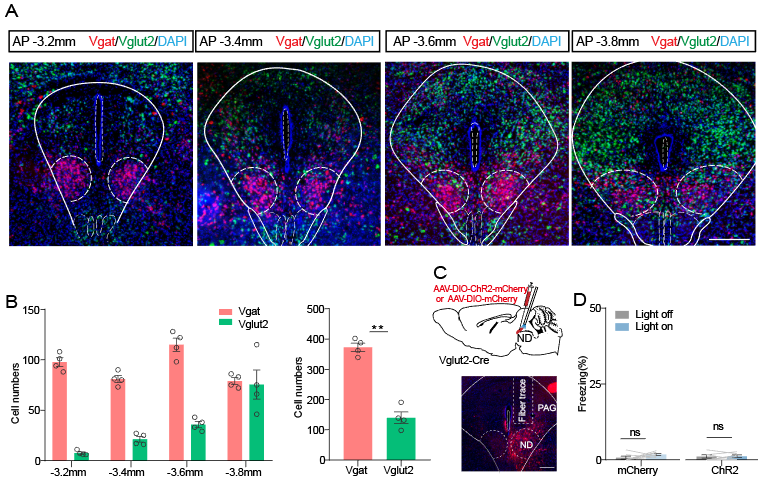


**Fig. S3 Distribution of GABAergic and glutamatergic cells in the ND and optogenetic activation of glutamatergic neurons in the ND.**

(A) Sample images showing the distributions of Vgat mRNA (red) and Vglut2 mRNA (green) in the ND.

(B) Quantitative number of Vgat mRNA (red) and Vglut2 mRNA (green)-expressing cells from anterior to posterior ND (left panel) and total cells number (right panel, n=4 mice, paired t-test, t=11.04, df=3, p=0.0016).

(C, D) Optogenetic activation of glutamatergic neurons in the ND (mCherry, n=7 mice, paired t-test, t=1.557, df=6, p=0.1704; ChR2, n=6 mice, paired t-test, t=0.1542, df=5, p= 0.8835).

Scale bar, 200 μm.**p*< 0.05, ***P*< 0.01, ****P*< 0.001. Data represent the mean ± SEM.

**Fig. S4**


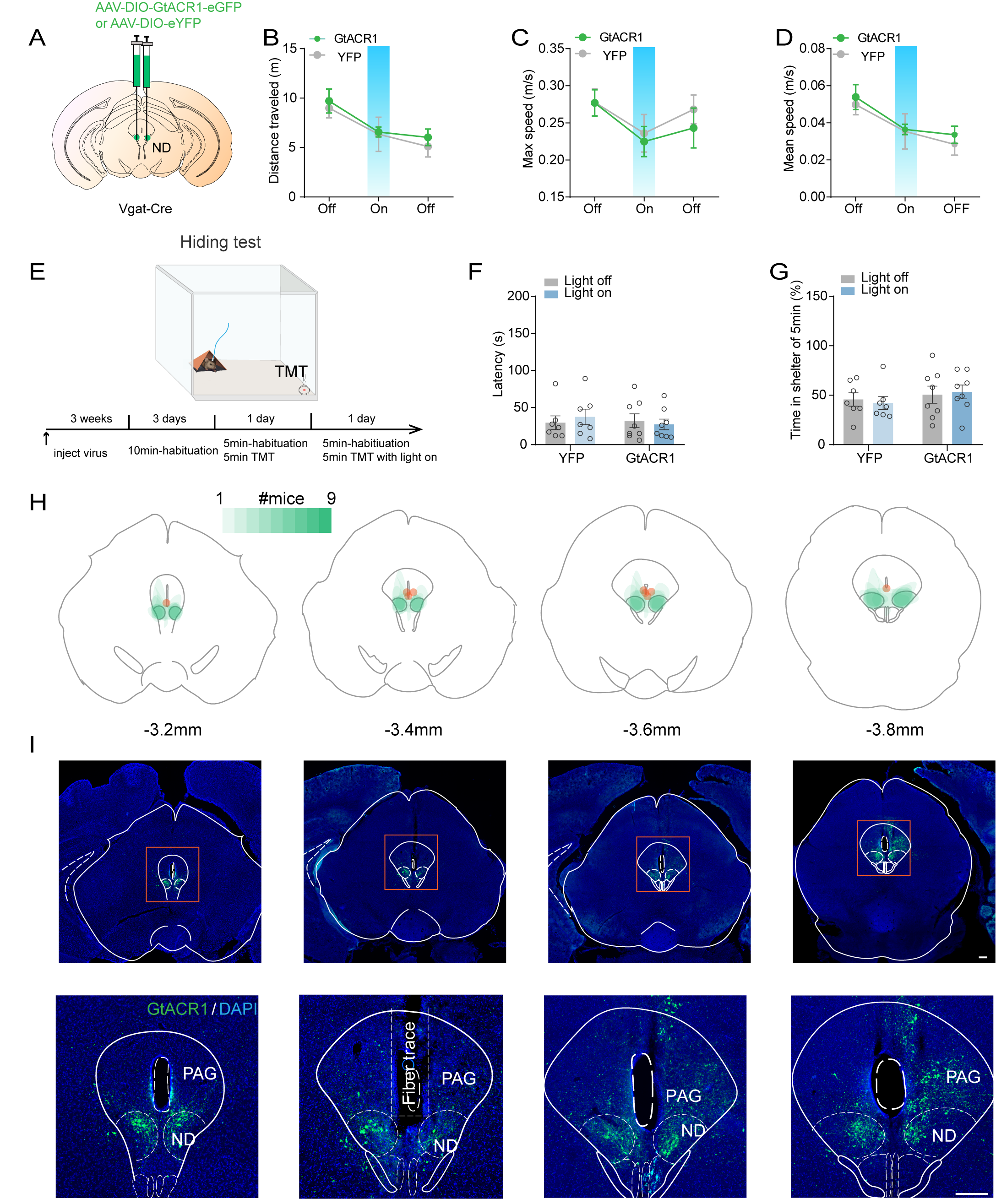


**Fig. S4 ND_GABA_ neurons inhibition did not change locomotion and hiding behavior.**

(A-D) Inhibition of ND_GABA_ neurons did not change travel distance and travel speed (GtACR1, n=9 mice; eYFP, n=6 mice, two-way repeated-measures ANOVA, B, F_(2, 26)_=0.1051, p=0.9006; C, F_(2, 26)_= 0.3318, p=0.7206; D, F_(2, 26)_=0.1191, p=0.8882).

(E-G) Inhibition of ND_GABA_ neurons had no significant effect on hiding behavior (GtACR1, n=8 mice; eYFP, n=7 mice, two-way repeated-measures ANOVA, F, F_(1, 13)_=0.6849, p=0.4228; G, F_(1, 13)_=0.4876, p=0.4973).

(H) Serial reconstruction of optical fiber implantation and viral injection sites in the ND.

(I) Coronal brain section samples show optical fiber implantation and viral injection sites.

Scale bar, 200 μm. Data represent the mean ± SEM.

**Fig. S5**


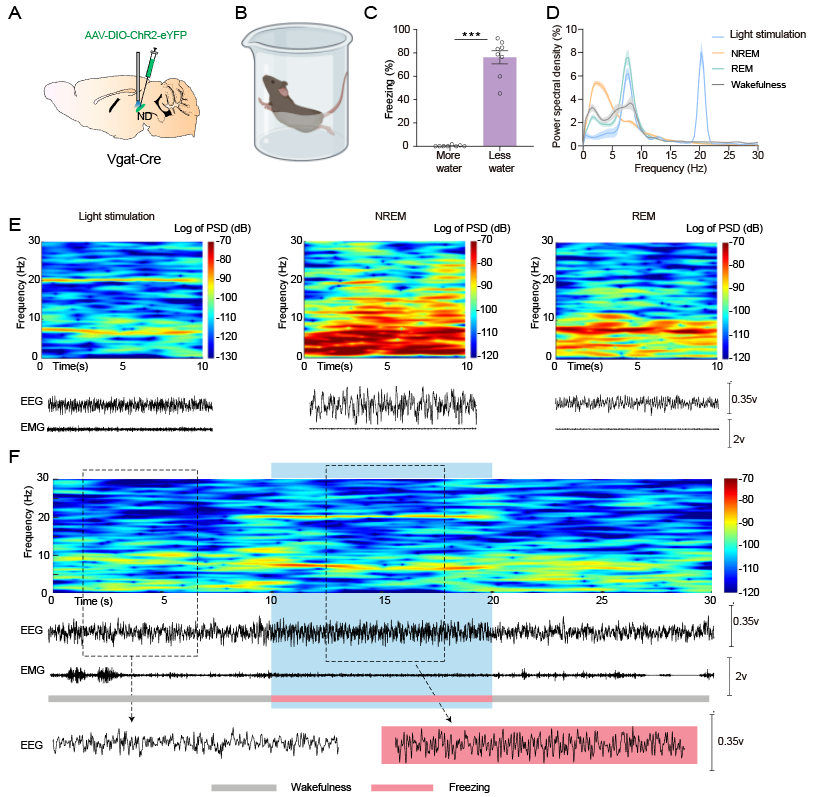


**Fig. S5 Optostimulation of ND_GABA_ neurons did not promote sleep or seizure response**

(A) Schematic for AAV-DIO-ChR2 injection and optical fiber implantation into ND.

(B) Experimental design of forced swimming test.

(C) Optogenetic activation of ND_GABA_ neurons induced freezing when a container with less water but not for more water (n=8 mice, paired t-test, t=13.57, df=7, p<0.0001).

(D) Relative cortical EEG power of light stimulation of ND_GABA_ neurons (blue trace), natural NREM (yellow trace), REM (green trace) and wakefulness (gray trace).

(E) Representative EEG power spectrogram, EEG trace, and EMG trace during light stimulation of ND_GABA_ neurons (left), NREM (middle), and REM (right).

(F) Representative EEG power spectrogram, EEG trace, and EMG trace during light stimulation of ND_GABA_ neurons and wake. The blue rectangle indicates the stimulation period, and the gray and red lines indicate wakefulness and freezing, respectively. ****p*< 0.001. Data represent the mean ± SEM.

**Fig. S6**


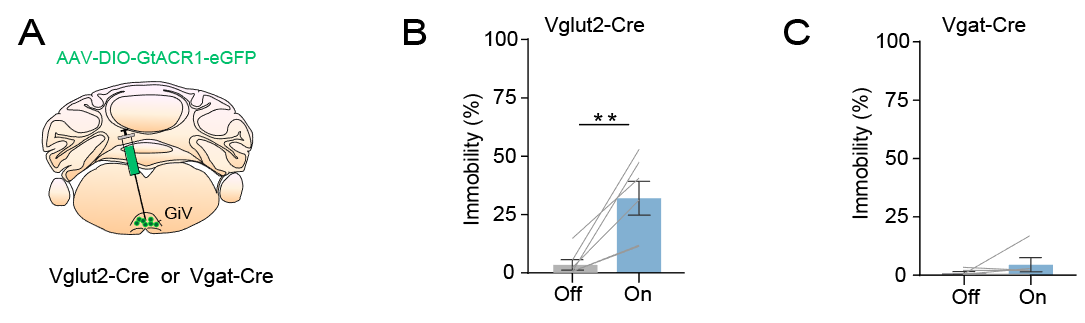


**Fig. S6 Optogenetic inhibition of the GiV_glu_ neurons induced freezing behavior.**

(A) Experimental design for Optogenetic inhibition of the GiV_glu_ or GiV_GABA_ neurons.

(B) Percentage of freezing evoked by light-stimulation of GiV_glu_ neurons (GtACR1, n=6 mice, paired t-test, t=4.312, df=5, p=0.0076).

(C) Percentage of freezing evoked by light-stimulation of GiV_GABA_ neurons (GtACR1, n=5 mice, paired t-test, t=1.071, df=4, p=0.3447).

Data represent the mean ± SEM.

**Graphical abstract**


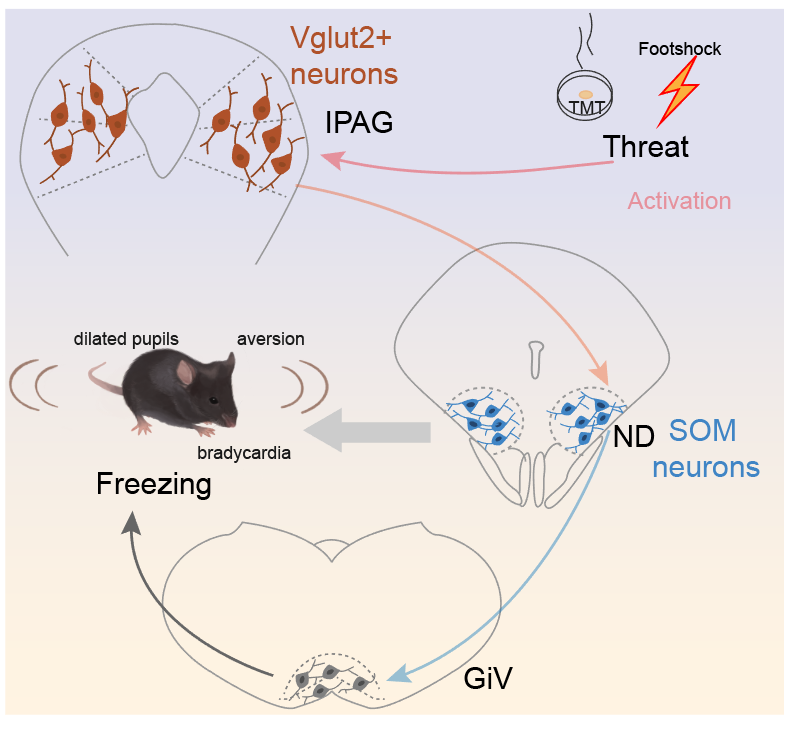


Schematic illustrating a neural circuit for ND_GABA_ relays fearful information from PAG to medulla to control freezing-like defensive behavior.

**Video S1:** Optogenetic excitation (20 Hz) of the ND_GABA_ neurons in Vgat-Cre mice induces immediate freezing-like behavior.

**Video S2:** Optogenetic excitation (20 Hz) of the ND_GABA_ neurons in Vgat-Cre mice has different responses in the modified forced swimming tests with different volumes of water.
