# Supplementary material for: Control of defensive behavior by the nucleus of Darkschewitsch GABAergic neurons
Source: Natl Sci Rev. 2024 Mar 5;11(4):nwae082. doi: 10.1093/nsr/nwae082 (PMC11057443; doi:10.1093/nsr/nwae082)
Supplement: nwae082_Supplemental_File [file nwae082_supplemental_file.zip › Supplementary Meterials and Methods.docx]

## METHOD DETAILS

**Stereotaxic surgeries and viral injections**

AAV2/9-hSyn-Flex-GCaMP6m-WPRE-SV40pA (6.7x10^12^ ppl/mL), AAV2/9-CAG-DIO-GtACR1-P2A-EGFP (5.5x10^12^ ppl/mL), AAV2/9-hEF1a-DIO-hChR2(H134R)-EYFP-WPRE-pA (3.05x10^12^ ppl/mL), scAAV2/2Retro-hSyn-Flpo-pA (6.05x10^12^ ppl/mL), scAAV2/1-hSyn-Cre-pA (5.01x10^12^ ppl/mL), scAAV2/2-hSyn-FLEX-Flpo-pA (6.45x10^12^ ppl/mL), AAV2/9- hEF1a-fDIO-hChR2(H134R)-eYFP-WPRE-pA (6.3x10^12^ ppl/mL), AAV2/9-hEF1a-fDIO-eYFP-WPRE-pA (6.43x10^12^ ppl/mL), AAV2/5-hSyn-DIO-eYFP (5x10^12^ ppl/mL), AAV2/9-hSyn-DIO-hM3D(Gq)-mCherry (4.5x10^12^ ppl/mL) and AAV2/9-hEF1a-DIO-eYFP-WPRE-pA (5.2x10^12^ ppl/mL) were prepared by Taitool Bioscience. The following viral vectors were prepared by BrainVTA: AAV2/9-CAG-DIO-TVA-eGFP (3.7x10^12^ ppl/mL), Rabies viruses ΔG-DsRed (EnvA) (5x10^8^ ppl/mL), AAV2/9-CAG-DIO-RVG (2.57x10^12^ ppl/mL). The following viral vectors were prepared by Addgene: pAAV5-hSyn-Con/Fon-hChR2(H134R)-eYFP (6.0x10^12^ ppl/mL), pAAV8-hSyn-Con/Fon-mCherry (4.5x10^12^ ppl/mL), pAAV5-EF1α-DIO-hChR2(H134R)-eYFP-WPRE-HGHpA (5x10^12^ ppl/mL). The following vectors were from OBIO: pAAV2/9-Ef1a-DIO-hChR2(H134R)-mCherry (6.3x10^12^ ppl/mL), pAAV2/9-hSyn-DIO -mCherry (4.9x10^12^ ppl/mL).

Mice were anesthetized with ketamine and xylazine (100 mg per kg body weight and 10 mg per kg body weight, i.p. injection) and placed in a stereotaxic instrument (RWD Life Science). Approximately 50-200 nL of AAV was injected unilaterally or bilaterally into the ND nucleus (50 nL; bregma: -3.50 mm, lateral: 0.25mm, ventral: -3.20 mm), GiV (100 nL; bregma: -6.6 mm, lateral: 0.5 mm, ventral: -5.65 mm), lPAG (150-200 nL; bregma: -4.75 mm, lateral: 0.5mm, ventral: -2.30 mm) using a syringe pump (KD Scientific) at a rate of 30 nL/min. The injection needle was withdrawn 10 min after the end of the infusion and the incision was closed with suture. For optical fiber implantation, mice received a chronically implanted optical fiber (diameter 200 μm, NA 0.37, Newdoon) dorsal to the ND nucleus (bregma: -3.50 mm, lateral: 0.25mm, ventral: -3.00 mm), GiV (bregma: -6.6 mm, lateral: 0.5 mm, ventral: -5.45 mm), lPAG (bregma: -4.75 mm, lateral: 0.5mm, ventral: -2.10 mm). Experiments were performed 3-6 weeks (for AAVs) and 7 days (for Rabies viruses) after stereotaxic injection.

**Behavioral assays**

***Freezing test***

The freezing test paradigm was performed using previously described methods[1], with some modifications. Briefly mice were put in a Plexiglas box (L x W x H: 30 cm x 20 cm x 30 cm) and after 1 min of habituation, 473 nm laser stimulation (20 Hz, 5 ms, 2.5-5 mW for neuronal cell bodies, 2-7.5 mW for projections) was applied six times for 10 s, with an inter-stimulus interval of 50 s. The average duration of freezing of six times during the ‘light on’ periods as the animal freezing time. All the trials were recorded by a USB camera attached to the computer and the time of freezing was analyzed offline using ANY-maze software 5.3.

***Fear conditioning***

The fear conditioning paradigm was performed as previously described methods[1], with some modifications. Briefly, mice were put in an auditory fear conditioning box (L x W x H: 14 cm x 14 cm x 20 cm) with a metal grid floor. After 2 min habituation, a 30 s tone (4 kHz, 85 dB) was used as the conditioned stimulus (CS) followed an electric foot shock (0.8 mA, 2 s duration) as the unconditioned stimulus (US), the conditioning session including four trials (120 s interval). On the day after conditioning, mice were exposed to four CS-only presentations in a dimly illuminated context different from the conditioning context. While the conditioning context was cleaned with 75% ethanol, the retrieval context was wiped down with 1% acetic acid before testing mice. The duration of the retrieval session was 630 s, with a baseline period of 120 s and a pseudo-random presentation of the CS with a minimal inter-stimulus interval of 90 s.

To test for ND_GABA_ neurons inhibition by light-stimulation of GtACR1 effects on CS-induced passive defensive behavior, the second and fourth presentation of the CS was paired with 30 s of continuous 473 nm light-stimulation (constant, 5 mW). The total duration of freezing during the two CS-alone periods was compared to the freezing time of CS-paired with light-stimulation and of the equivalent baseline period (60 s). All events in the fear conditioning test were programmed, and data were recorded through the MED software (MED Associate Inc). To test

***Predator odor evoked freezing***

Mice were directly placed in a Plexiglas box (L x W x H: 30 cm x 20 cm x 30 cm) with a TMT odor dish in one corner. Five 15 s cycles of blue light (constant, 5 mW) were delivered to the ND while the mice were immobile. The percentage freezing time was calculated for 15 s laser off, laser on, and laser off periods, and the values for all stimulations were averaged to give the freezing level for each mouse.

***Looming test***

Freezing behavior triggered by looming visual stimuli were measured in an arena (L x W x H: 40 cm x 40 cm x 30 cm). No shelter nest was placed in the arena. The looming stimulus was a 1 cm (thus a visual angle of diameter 2° when directly over the animal) black disk rapidly widening to 21.8 cm (20°) in 250 ms and remaining on the screen at this size for an additional 50 ms, and was repeated 15 times. This together with a 30 ms pause between each repeat, so the total looming visual stimuli last 4.95 s. Behavior was recorded using an HD digital camera (Logitech). Animals were handled one day before testing. During the looming test session, mice were first allowed to freely explore the looming box for 5~10 min, then received the looming stimulus. Looming stimuli were performed four times at about 1~2 min intervals and the first and third presentation of the looming stimuli were paired with 473 nm light-stimulation (constant, 5 mW). Light stimulation was delivered about 1 s before onset of the looming stimulus and continued until the looming was turned off.

***Heart rate measurements***

Heart rate was measured with pulse oximetry (MouseOx Plus; Starr Life Sciences, Alllison Park, PA, USA). Mice was shaved in the area surrounding the neck and acclimated to moving with the collar sensor used by the pulse oximeter for two days. Eventually, 10 min of baseline was recorded using the software (Conscious Software Module, Starr Life 782 Sciences), after which the mouse received 1 min of photo-stimulation (20 Hz, 5ms) followed by 1 min of post-stimulation measurements. Heart rate was recorded as a moving average of 3-5 measurements recorded at 1 Hz.

***Pupil size measurements***

An infrared-sensitive camera was used to capture the pupil image under when the animals were head-fixed. Each trial lasted 45 s. Light (473 nm) was delivered to the ND at 20 Hz, 5ms pulses for 15 s following a 15 s baseline recording and another 15 s were recorded post-stimulation (3 times each). After acquiring the video, we detected the pupil in each frame and calculated normalized pupil diameter using a custom-written MATLAB script.

***Conditioned place aversion (CPA) test***

The CPA test consisted of 5 sessions over 5 days and was performed in a classical CPA chamber as described in previous study[2]. On day 1, individual mouse was placed in the center of the box and allowed to freely explore the entire apparatus for 15 min (pre-test). On days 2-4, mice were confined to one of the side chambers (conditioned compartment) for 30 min light-excitation (473 nm, 20 Hz, 5 ms, repeated 30 s laser on with 30 s intervals) of ND_GABA_ neurons. Five hours later, mice were placed in the opposite side chamber for another 30 min without light-stimulation (unpaired compartment). On day 5, mice were placed in the middle compartment and allowed to freely explore the entire apparatus for 15 min (post-test) The time spent in the conditioned compartment during the pre-test was compared with the time spent in the same compartment during the post-test was automatically calculated with the ANY-maze software 5.3 via a webcam (Logitech web-camera).

***Open field test***

Mice were placed in an open field arena (L x W x H: 50 x 50 x 60 cm). For chemogenetic activation of the ND_GABA_ neurons, mice were allowed to freely explore for 5 min. For optogenetic inhibition of the ND_GABA_ neurons, mice were allowed to freely explore for 9 min, the 9 min session was divided into three sessions consisting of 3 min laser off, 3 min laser on, and 3 min laser off. The total locomotion activity in 5 min or 9 min was analyzed using the ANY-maze software 5.3.

***Hiding test***

The hiding test paradigm was performed using previously described methods[3, 4], with some modifications. For optogenetic activation of the ND in Fig. 3D, mice were placed into an open field arena (L x W x H: 50 x 50 x 60 cm) with a shelter (L x W x H: 14 x 11 x 9 cm) on the corner. Mice were placed in the test box 3 days (10 min per day) for habituation. During the testing day mice were first allowed to freely explore the testing box for 3-5 mins, they received optogenetic manipulation (20 Hz, 5 ms) for 20 s while they were not in the shelter. We analyzed (1) latency to return to shelter: the time from photo-stimulation presentation to time when animal entered the shelter; (2) time spent in the shelter (% of 2 mins bin): time spent in the shelter following photo-stimulation onset. For optogenetic inhibition of the ND_GABA_ neurons in Fig. S4E-G, mice were placed in the testing box for a 5 min habituation period with an empty dish (on the first test day), followed by a 5 min test period without light stimulation but the mice were exposed to TMT (2.5 uL). On the second test day, the protocol was the same as the first day but 473 nm light stimulation (constant light, 5 mW) was applied. The latency to return to the shelter and the total time spent in the shelter were analyzed.

***Electroencephalogram (EEG) and Electromyography (EMG) Recording***

EEG signals were recorded from electrodes on the frontal cortices (bregma: 2 mm; lateral: 1 mm). Two stainless-steel wires were inserted into neck muscles as EMG electrodes. The EEG electrodes, optical fiber, and EMG electrodes were fixed to the skull with dental cement. The animal was kept on a heating pad until fully recovered from anesthesia. After 1 week of surgery recovery, the animal was placed in a sound attenuated recording box and allowed to habituate the recording box for 3 days. For stimulation, the optical fiber and EEG recording cable were connected to a 473 nm laser diode and EEG recording device through a slip-ring device (CFS-22) and fiber-optic rotary joints (Doric Lenses) to avoid the EEG recording cable and optic fiber tangling. The EEG and EMG signals derived from the implanted electrodes were amplified, filtered (EEG, bandpass filter, 0.5-50Hz; EMG, high-pass filter,10Hz) by AC amplifier (Model 1700, A-M Systems), digitized at 200 Hz by PowerLab (ML795, AD Instruments), and recorded by LabChart software (AD Instruments). The sleep analysis software SleepSign (Kissei Comtec) was used for sleep state scoring. All scoring was automatic on the basis of the EEG and EMG waveforms for each 4 s epoch. Sleep state was defined as follows. Wakefulness: desynchronized EEG and high EMG activity; NREM sleep: synchronized EEG with high power at delta frequencies (0.5-4 Hz) and low EMG activity; REM sleep: desynchronized EEG with high power at theta frequencies (4-10 Hz) and low EMG activity. The EEG power spectral density analysis was done with NeuroExplorer (Plexon). To normalize the data, we used the relative EEG power represented by the ratio of the power spectral density in the different frequency ranges to the average value of total power in the same epoch. To compare the EEG spectrum of photo-stimulation induced freezing with NREM and REM, we analyzed the relative EEG power during natural NREM, REM and photo-stimulation induced freezing with a 10 s duration in a 0.5-30 Hz window with 0.38 Hz resolution.

***Modified forced swimming test***

To investigate whether mice had ability to escape from danger when ND_GABA_ neurons were excited, we performed the modified forced-swimming test. ND_GABA_ ChR2-expressing mice were placed in a 1 L beaker filled with 500 mL (shallow water, mice can stand at the bottom with hind paws) or 800 mL (deep water, mice floating in the water) water (25 ℃) and 10 s 473 nm laser (20 Hz, 5ms) was delivered to activate ND_GABA_ neurons. The immobility time was recorded by a video camera and calculated manually with a stopwatch. The test was repeated three times for each mouse and the average of immobility time as the mice freezing time.

**RNAscope *in situ* hybridization**

RNAscope Fluorescent Multiplex Assays with the Slc32a1 probe, Slc17a6 probe, Sst probe and Pvalb probe were used to determine the overlap of different neuron types in the ND. Brain sections (40 μm) from Vgat-venus mice or C57/Bl6J containing ND nucleus were collected with a cryostat (Leica CM 1950), and stored in DepC-PBS. Floating sections were mounted on glass slides, heated at 60 °C for 2 h, and kept at -80 °C before experiments. Slides were treated thrice with ethanol (5 min each time) and air-dried at room temperature. Then, the slides were pretreated with hydrogen peroxide at room temperature for 10 min and washed twice in DepC-PBS (2 min each time). Protease digestion was performed in a 40 °C HybEZ oven for 15 min. After washing in DepC-PBS for 3 min and rinsing in DepC-ddH_2_O, slides from C57 mice were hybridized with pre-warmed Slc32a1 probe (Vgat), Slc17a6 probe (VgluT2), slides from Vgat-venus mice were hybridized with pre-warmed Sst probe (SOM), Pvalb probe (PV), positive control probe (peptidylprolyl isomerase B, Ppib), or negative control probe [dihydrodipicolinate reductase (dapB) gene] in the 40 °C HybEZ oven for 2 h. The signal amplification fluorescent label was TSA-based. RNAscope multiplex fluorescent reagent kit v2 and appropriately designed probes (ACDBio Inc.) were used. The probes were alternated across all sections to ensure that one posterior section and one anterior section from each region were analyzed with each probe type.

**Rabies viruses (RV) tract tracing procedure**

The modified RV-based monosynaptic retrograde tracing approach was used for mapping the whole-brain inputs onto ND_GABA_ neurons. A mixture of AAV2/9-CAG-DIO-eGFP-2A-TVA and AAV2/9-CAG-DIO-RVG (1:1, 30-40 nL) helper viruses was stereotaxically injected into the ND of Vgat-IRES-Cre mice. Two weeks after AAV helper viruses injection, RV-ENVA-ΔG-DsRed (30-40 nL) was injected into the same location in the ND of Vgat-IRES-Cre mice. One week after RV injection, mice were perfused with saline followed by 4% PFA in PBS. Forty μm coronal brain sections were prepared using a cryostat (Leica CM 1950). The coronal brain sections were imaged with an Olympus VS120 epifluorescence microscope (x10 objective) and analyzed with ImageJ (National Institutes of Health and Laboratory for Optical and Computational Instrumentation, University of Wisconsin, USA). Only starter cells (coexpression of TVA-eGFP and RV-DsRed) restricted in the ND samples were included in our study. Only brain areas with at least ten labeled neurons were analyzed. The cell number in each brain area was normalized by total RV-labeled cell number as for the fraction of total cells.

**Anterograde tract tracing**

We injected 50 nL pAAV5-hSyn-DIO-eYFP into the ND of Vgat-Cre mice. For quantification of fluorescence intensities, confocal images were acquired using a 20x objective with identical pinhole, gain, and laser settings. For each brain region, 3-5 images at the same focal level from different sections were collected from each animal. No additional post-processing was performed on any of the collected images. The fluorescence intensity for eYFP expression was then quantified in each region as percentage per pixel using ImageJ software.

**Fos protein immunostaining**

Before applying foot shocks or TMT, mice were given 7 days to habituate the experimental test environment. For the foot shocks test, mice were subjected to experimental apparatus (L x W x H: 14 cm x 14 cm x 20 cm) with a metal grid floor. After 2-min habituation, four shocks (0.8 mA, 2s duration) were directly delivered to mice, with a 90 s inter-trial interval. The control group was treated as the experiment group except for foot shock (0 mA, 2 s duration). For TMT exposure, a dish with 2.5 uL TMT was placed on the corner of the experimental apparatus made of clear Plexiglas box (L x W x H: 30 cm x 20 cm x 30 cm). Mice were subjected to the experimental apparatus for 15 min. The control group was treated as the experiment group instead of TMT but 0.9% saline. After stimulation, all mice were back to homecage and 1.5 h later, the animals were deeply anesthetized with sodium pentobarbital (40 mg/kg, i.p.), and perfused with saline followed by 4% PFA in 0.1 M PBS, pH 7.4. Brains were removed, post-fixed overnight in 4% PFA at 4 °C and transferred to 30% sucrose in 0.01 M PBS, pH 7.4. Coronal sections (40 μm) were cut and stored in 0.01 M PBS. The sections were incubated in blocking buffer containing 3% bovine serum albumin and 5% normal goat serum in 0.2% Triton X-100/PBS (PBST) for 1 h at room temperature and then with primary antibodies in blocking buffer overnight at 4 °C. The primary antibodies used were Fos (1:1000, rabbit, sysy 226003). After three times rinsing with 0.01 M PBS, sections were incubated with fluorescent secondary antibodies at room temperature for 1 h. After another three times rinsing in 0.01 M PBS, sections were incubated with DAPI for 5 min, rinsed several times and then mounted on Prolong anti-fade medium (Invitrogen), with immunofluorescence assessed using a laser confocal microscope (Olympus FV-3000). Cell counting was carried out manually using ImageJ.

**Fiber photometry**

Calcium transients were measured using a fiber photometry system (Thinker Tech Nanjing Biotech Ltd, Nanjing, China). To minimize GCaMP6m bleaching, the laser power at the tip of the optical fiber was adjusted to a low level (0.03-0.04 mW). The GCaMP6 fluorescence was filtered with an eYFP bandpass filter and collected by a photomultiplier tube (R3896; Hamamatsu Photonics). An amplifier converted the photomultiplier current output to a voltage signal, which was further filtered through a low-pass filter (40 Hz cut-off; Brownlee 440). The analog voltage signals were digitized at 500 Hz (Power 1401 digitizer, Cambridge Electronic Design) and sampled with software (TDMS). Fiber-photometric recording data were acquired using custom acquisition code written in MATLAB (R2018a). For fiber photometry recordings in Fig. 1H regarding foot shocks, the mice were placed on the mental grid floor and received four 0.8 mA shocks (0.8 mA each, 2 s duration, 90 s intervals between shocks). For fiber photometry recordings in Fig. 1G, I and J regarding TMT, lemon odor and 1% acetic acid, a dish with TMT or lemon juice was gently delivered to the noses of the mice (5 s duration, 4-6 trials, 30-60 s intervals). Experimental time stamps were acquired using TTL pulses generated by a manual pulse generator which were then synchronized to calcium recordings at the designated time during the recording. The fluorescence change values (dF/F) were calculated as (F–F_0_)/(F_0_–V_offset_), where F_0_ is the baseline fluorescence signal averaged over a 2 s time-window prior to a trigger event and V_offset_ is the fluorescence signal recorded before the cannula was connected to the optical fiber above the ND. The dF/F values were presented as heatmaps or average plots with a shaded area indicating the SEM.

***In vivo* electrophysiology**

Animals were implanted with a self-made stereotrodes[5] above the ND (bregma: -3.5 mm, lateral: 0.25 mm, ventral: -3.0 mm), which consisted of 32 single microwires (California Fine Wire Co., Grover Beach, CA, USA). One ground wire was soldered to a 32-channel connector (Omnetics Connector Corp., Minneapolis, MN, USA). Mice were allowed to recover for at least one week, and then the electrodes were connected to a 32-channel preamplifier head-stage (Plexon Inc., Dallas, TX, USA). During the recording sessions, all signals recorded from each electrode were amplified, filtered between 0.1 Hz and 8 kHz, and spike waveforms were digitized at 40 kHz. Spikes were sorted using the software Offline Sorter (Plexon). Units were accepted only if a distinct cluster was visible in a two-dimensional plot of the largest two principal components. In total, 3 mice were implanted with electrodes and used for data collection. At the end of each recording session, the stereotrode was moved ventrally for ~50 μm. For freezing behavior induced by TMT, mice were placed in a Plexiglas box (L x W x H: 30 cm x 20 cm x 30 cm) with a TMT odor dish in one corner and behaviors were recorded using an HD digital camera (Logitech). We analyzed the video frame-by-frame (60 frames/sec) with professional video editing software (Adobe Premiere Pro CC 2018) to recognize the moment when the mouse sniffing the dish of TMT (mouse’s nose was just touching the dish). The period of half-second before and after this moment was determined as the TMT period. The experimental time stamps were acquired using a manual TTL pulse generator. A given neuron that significantly responded to a defined behavior would be reflected by its reliable responses across different trials. The responses of each neuron were averages of 2-13 trials (for approaching/sniffing TMT) or 10-49 behavioral trials (for freezing). We then calculated the averaged response during approaching/sniffing TMT and freezing, based on the Z-scored PSTHs (peri-stimulus time histogram)[6]. PSTH(t) for approaching TMT, was calculated with 100-ms resolution between t = -1 s to t = 1 s relative to approaching TMT onset for each cell. PSTH(t) for freezing, was calculated with 50-ms resolution between t = -1 s to t = 3 s relative to freezing onset for each cell. The mean (μ) and standard deviation (σ) of the firing rate preceding behaviors (t = -1 s to t = 0 s) were calculated and used to generate the *Z*-score normalized behavior-triggered PSTH:$Z\left( t \right)=\frac{\mathrm{PSTH}\left( t \right)-\mu}{\sigma}$. To determine behaviors (sniffing/approaching TMT and freezing) altered neural activity patterns (that is, excitation, inhibition or no response), units were defined as excitation or inhibition if the average *Z*-score between t=0 s and t=1 s (for approaching/sniffing TMT) or t=3s (for freezing) exceeded ±1.96 (corresponding to p < 0.05, corrected for multiple comparisons). In particular, in the heatmap of Fig. 2H, if *Z*-score >12, then we defined it equals 12.

## QUANTIFICATION AND STATISTICAL ANALYSIS

Data are presented as means + SEM. For comparisons with only two groups, P values were calculated using paired or unpaired t-tests as described in the figure legends. Comparisons across more than two groups were made using a one-way ANOVA, and a two-way ANOVA was used when there was more than one independent variable. A Tukey or Bonferroni post-test was used following significance with ANOVA. * *p*<0.05, ** *p*<0.01, *** *p*<0.001. No data were excluded from the analyses and experimental and control animals were randomized throughout the study. Data from fiber photometry were analyzed by MATLAB. Sleep analysis were achieved by NeuroExplorer and MATLAB. All statistical graphs and significant analysis were made by Graphpad Prism 8.

## REFERENCES

1. Tovote P, Esposito MS, Botta P *et al.* Midbrain circuits for defensive behaviour. *Nature*. 2016; **534**(7606): 206-+. doi: 10.1038/nature17996

2. Yang HB, de Jong JW, Cerniauskas I *et al.* Pain modulates dopamine neurons via a spinal-parabrachial-mesencephalic circuit. *Nat Neurosci*. 2021; **24**(10): 1402-1413. doi: 10.1038/s41593-021-00903-8

3. Zhou Z, Liu X, Chen S *et al.* A VTA GABAergic Neural Circuit Mediates Visually Evoked Innate Defensive Responses. *Neuron*. 2019; **103**(3): 473-488 e476. doi: 10.1016/j.neuron.2019.05.027

4. Miller SM, Marcotulli D, Shen A *et al.* Divergent medial amygdala projections regulate approach-avoidance conflict behavior. *Nat Neurosci*. 2019; **22**(4): 565-575. doi: 10.1038/s41593-019-0337-z

5. Lin L, Chen G, Kuang H *et al.* Neural encoding of the concept of nest in the mouse brain. *Proc Natl Acad Sci U S A*. 2007; **104**(14): 6066-6071. doi: 10.1073/pnas.0701106104

6. Wolff SB, Grundemann J, Tovote P *et al.* Amygdala interneuron subtypes control fear learning through disinhibition. *Nature*. 2014; **509**(7501): 453-458. doi: 10.1038/nature13258
