# Supplementary material for: Control of defensive behavior by the nucleus of Darkschewitsch GABAergic neurons
Source: Natl Sci Rev. 2024 Mar 5;11(4):nwae082. doi: 10.1093/nsr/nwae082 (PMC11057443; doi:10.1093/nsr/nwae082)
Supplement: nwae082_Supplemental_File [file nwae082_supplemental_file.zip › Supplementary Table 1.pdf]

Supplementary Table 1. Summary of statistical analysis

Figure	Mean±SEM	Sample size (n)	Statistical test	P values
Fig.1B	Fos cells:<br>Ctrl:20±2<br>TMT:165±17<br>Vgat+Fos+ cells:<br>Ctrl:5±1<br>TMT:128±24<br>Vgat+Fos+/Fos+(%):<br>Ctrl:25.38±2.32<br>TMT:76.27±6.95<br>Vgat+Fos+/Vgat+(%):<br>Ctrl:1.18±0.15<br>TMT:31.43±7.7	Ctrl: 4 mice<br>TMT: 3 mice	two-tailed unpaired t test;<br>two-tailed unpaired t test;<br>two-tailed unpaired t test;<br>two-tailed unpaired t test	P=0.0002,t=10.24, df=5;<br>P=0.0016,t=6.156, df=5;<br>P=0.0005,t=7.916, df=5;<br>P=0.0054,t=4.696, df=5
Fig.1D	Fos cells:<br>Ctrl:30±5<br>Shock:158±17<br>Vgat+cfos+ cells:<br>Ctrl:11±5<br>Shock:111±15<br>Vgat+Fos+/Fos+(%):<br>Ctrl:33.2±10.46<br>Shock:69.9±4.12<br>Vgat+Fos+/Vgat+(%):<br>Ctrl:2.56±1.14<br>Shock:27.57±4.35	Ctrl: 3 mice<br>Footshock: 3 mice	two-tailed unpaired t test;<br>two-tailed unpaired t test;<br>two-tailed unpaired t test;<br>two-tailed unpaired t test	P=0.002,t=7.133, df=4;<br>P=0.0034,t=6.224, df=4;<br>P= 0.0309,t=3.265, df=4;<br>P=0.0051,t=5.566, df=4
Fig.1G	-5-0s:-1.13±2.7<br>0-5s:48.02±3.81	3 mice	two-tailed paired t test	P =0.0033,t=17.33, df=2
Fig.1H	-5-0s:1.11±3.24<br>0-5s:97.61±18.9	7 mice	two-tailed paired t test	P =0.0013,t=5.653, df=6
Fig.1I	-5-0s:4±1.31<br>0-5s:-1.77±0.47	3 mice	two-tailed paired t test	P =0.0809,t=3.299, df=2
Fig.1J	-5-0s:0.83±4.37<br>0-5s:1.5±1.91	3 mice	two-tailed paired t test	P =0.9114,t=0.1258, df=2
Fig.2F	Pre:20.93±6.26<br>TMT:36.39±8.69	16 neurons	two-tailed paired t test	P =0.0002,t=4.787, df=15
Fig.2I	Pre:14.89±4.8<br>Freezing:22.21±5.75	12 neurons	two-tailed paired t test	P =0.0115,t=3.030, df=11
Fig.3B	YFP:<br>Light off:2.5±2.18<br>Light on:2.78±0.93<br>ChR2:<br>Light off:1.33±0.91<br>Light on:88.49±3.54	YFP : 6 mice<br>ChR2 : 8 mice	YFP:two-tailed paired t test;<br>ChR2:two-tailed paired t test	P=0.9177,t=0.1087, df=5;<br>P<0.0001,t=26.67, df=7
Fig.3D	Latency(s):<br>YFP:43.94±10.19<br>ChR2:52.96±8.97<br>Time in nest(%):<br>YFP:27.96±6.36<br>ChR2:39.36±8.92	YFP : 6 mice<br>ChR2 : 6 mice	two-tailed unpaired t test;<br>two-tailed unpaired t test	P=0.5160,t=0.6643, df=16;<br>P=0.3138,t=1.040, df=16
Fig.3E	YFP:<br>Off:1±0<br>On:1.08±0.12<br>Off:0.93±0.06<br>ChR2:<br>Off:1±0<br>On:2.11±0.15<br>Off:1.63±0.17	YFP : 6 mice<br>ChR2 : 6 mice	Both:one-way repeated-measures ANOVA factor: treatment(before,on,after)<br>Turkey's multiple comparisons test	(left)YFP:treatment: F (1.406, 7.030) = 1.280, P =0.3168<br>multiple comparisons:<br>before vs on, P = 0.7851<br>before vs after, P =0.5289<br>on vs after, P =0.3333<br>(right)ChR2:treatment: F (1.957, 9.785) = 25.95, P =0.0001<br>multiple comparisons:<br>before vs on,P=0.0017<br>before vs after,P= 0.0283<br>on vs after,P=0.0480

Fig.3F	YFP:<br>Off:702.3±8.46<br>On:698.3±10.11<br>Off:693.4±10.63<br>ChR2:<br>Off:706.6±17.19<br>On:598.6±27.86<br>Off:654.6±20.5	YFP : 6mice<br>ChR2 : 6mice	Both:one-way repeated-measures ANOVA factor: treatment(before,on,after) Turkey's multiple comparisons test	(left)YFP:treatment: F (1.812, 9.061) = 0.4136, P=0.6542<br>multiple comparisons:<br>before vs on, P= 0.8986<br>before vs after, P= 0.7246<br>on vs after,P=0.8522<br>(right)ChR2:treatment: F (1.367, 6.834) = 11.91, P=0.0084<br>multiple comparisons:<br>before vs on, P = 0.0203<br>before vs after, P = 0.0204<br>on vs after, P =0.1588
Fig.3G	YFP:<br>pre:53.06±1.37<br>post:55.55±1.56<br>ChR2:<br>pre:60.32±3.56<br>post:45.62±3.83	YFP : 5 mice<br>ChR2 : 5mice	two-way repeated-measures ANOVA factor one: virus (YFP, ChR2)<br>factor two: laser(Pre, Post)<br>Bonferroni's multiple comparisons test	virus: F (1, 8) = 0.3179 P = 0.5883<br>laser: F (1, 8) = 3.645 P = 0.0926<br>interaction: F (1, 8) = 7.223, P = 0.0276<br>multiple comparisons:<br>Pre:YFP vs. Pre:ChR2 , P=0.1735<br>Post:YFP vs. Post:ChR2, P =0.0478<br>YFP: Pre vs. YFP: Post, P > 0.9999<br>ChR2: Pre vs. ChR2: Post, P =0.0234
Fig.3J	YFP<br>Off:92.73±1.19<br>On:87.99±2.56<br>Off:90.61±2.21<br>GtACR1<br>Off:82.4±3.06<br>On:35.35±3.6<br>Off:80.5±1.86	YFP : 6 mice<br>GtACR1 : 9mice	Both:one-way repeated-measures ANOVA factor: treatment(before,on,after) Turkey's multiple comparisons test	(left)YFP:treatment: F (1.501, 7.507) = 2.712, P=0.1357<br>multiple comparisons:<br>before vs on, P =0.1641<br>before vs after, P = 0.3482<br>on vs after, P =0.5684<br>(right)GtACR1 : treatment:F (1.818, 14.55) = 74.30,P<0.0001<br>multiple comparisons:<br>before vs on, P <0.0001<br>before vs after, P = 0.8674<br>on vs after, P <0.0001
Fig.3L	YFP<br>CS:87.84±3.13<br>CS+light:88.72±6.3<br>GtACR1<br>CS:91.93±2.76<br>CS+light:25.44±6.77	YFP : 6 mice<br>ChR2 : 8mice	YFP:two-tailed paired t test;<br>GtACR1:two-tailed paired t test	YFP: P=0.8206,t=0.2390, df=5;<br>GtACR1:P =0.0001,t=7.559, df=7
Fig.3O	YFP:<br>On:58.6±5.969<br>Off:52.8±3.768<br>GtACR1;<br>On:10.29±6.669<br>Off:44.82±6.276	YFP : 7mice<br>GtACR1: 8mice	two-way repeated-measures ANOVA factor one: virus (YFP, GtACR1)<br>factor two: laser(on,off)<br>Bonferroni's multiple comparisons test	virus: F (1, 13) = 12.22,P=0.0039<br>laser: F (1, 13) = 40.23,P<0.0001<br>interaction:F (1, 13) = 79.28,P<0.0001<br>multiple comparisons:<br>on:YFP vs. on:GtACR1, P <0.0001<br>off:YFP vs. off:GtACR1 , P=0.0.5762<br>YFP: on vs. YFP: on, P=0.2061<br>GtACR1: off vs. GtACR1: on, P<0.0001
Fig.4D	YFP:<br>Light off:1.14±0.75<br>Light on:2.25±0.75<br>ChR2:<br>Light off:2.68±1.39<br>Light on:88.2±8.46	YFP : 7 mice<br>ChR2 : 7mice	YFP:two-tailed paired t test;<br>ChR2:two-tailed paired t test	P=0.3492,t=1.015, df=6;<br>P<0.0001,t=10.59, df=6
Fig.4E	YFP:<br>Light off:8.96±7.72<br>Light on:8.13±5.66<br>ChR2:<br>Light off:13.6±7.17<br>Light on:73.48±10.78	YFP : 5 mice<br>ChR2 : 6mice	YFP:two-tailed paired t test;<br>ChR2:two-tailed paired t test	P=0.7390,t=0.3573, df=4;<br>P=0.0011,t=6.709, df=5

Fig.5E	YFP:<br>Light off:3.4±1.25<br>Light on:4.19±0.32<br>ChR2:<br>Light off:3.81±1.08<br>Light on:84.7±4.53	YFP : 5 mice<br>ChR2 : 7mice	YFP:two-tailed paired t test;<br>ChR2:two-tailed paired t test	P=0.5432,t=0.6637, df=4;<br>P<0.0001,t=15.45, df=6
Fig.5F	YFP:<br>Light off:2.61±1.61<br>Light on:4.5±1.32<br>ChR2<br>Light off:1.13±0.59<br>Light on:69.8±5.5	YFP : 6 mice<br>ChR2 : 8mice	YFP:two-tailed paired t test;<br>ChR2:two-tailed paired t test	P=0.3807,t=0.9611, df=5;<br>P<0.0001,t=12.96, df=7
Fig.5G	mCherry:<br>Light off:3.94±0.76<br>Light on:5.87±1.26<br>ChR2:<br>Light off:4.18±1.13<br>Light on:73.9±6.84	mCherry : 6 mice<br>ChR2 : 8mice	mCherry:two-tailed paired t test;<br>ChR2:two-tailed paired t test	P=0.1471,t=1.715, df=5;<br>P<0.0001,t=11.19, df=7
Fig.6D	YFP:<br>Light off:1.19±0.81<br>Light on:1.35±0.63<br>ChR2:<br>Light off:1.04±0.52<br>Light on:1.34±0.44	YFP : 5 mice<br>ChR2 : 7mice	YFP:two-tailed paired t test;<br>ChR2:two-tailed paired t test	P=0.8540,t=0.1963, df=4;<br>P=0.7513,t=0.3318, df=6
Fig.6E	YFP :<br>Off:84.57±1.6<br>On:87.13±1.36<br>Off:83.11±3.31<br>ChR2:<br>Off:83.09±4.62<br>On:78.84±4.84<br>Off:73.85±3.43	YFP : 5 mice<br>ChR2 : 6mice	Both YFP and ChR2:one-way repeated-measures ANOVA<br>factor:<br>treatment(before,on,after)<br>Turkey's multiple comparisons test	YFP:treatment:F (1.709, 6.836) = 1.219, P=0.34347<br>multiple comparisons:<br>before vs on, P=0.5022<br>before vs after, P = 0.8466<br>on vs after, P =0.4561<br>ChR2:treatment:F (1.646, 8.230) = 3.365, P=0.0910<br>multiple comparisons:<br>before vs on, P =0.3739<br>before vs after, P = 0.1121<br>on vs after, P =0.3739
Fig.6F	YFP:<br>CS:88.42±2.54<br>CS+light:85.33±4.63<br>ChR2:<br>CS:92.96±0.97<br>CS+light:94.35±1.77	YFP : 5 mice<br>ChR2 : 6mice	YFP:two-tailed paired t test;<br>ChR2:two-tailed paired t test	YFP: P=0.4146,t=0.9093, df=4<br>ChR2:P =0.4272,t=0.8638, df=5
Fig.6H	YFP:<br>Light off:2.67±1.25<br>Light on:4.67±0.97<br>ChR2:<br>Light off:6.88±4.03<br>Light on:89.23±3.31	YFP : 5 mice<br>ChR2 : 9mice	YFP:two-tailed paired t test;<br>ChR2:two-tailed paired t test	P=0.3883,t=0.9671, df=4;<br>P<0.0001,t=15.49, df=8
Fig.6I	YFP:<br>Off:1±0<br>On:0.97±0.1<br>Off:1.03±0.16<br>ChR2:Off:1±0<br>On:1.76±0.12<br>Off:1.41±0.22	YFP : 5 mice<br>ChR2 : 7 mice	Both YFP and ChR2:one-way repeated-measures ANOVA<br>factor:<br>treatment(before,on,after)<br>Turkey's multiple comparisons test	YFP:treatment:F (1.572, 6.287) = 0.1149, P=0.8488<br>multiple comparisons:<br>before vs on, P =0.9552<br>before vs after, P = 09552<br>on vs after, P =0.9552<br>ChR2:treatment:F (1.466, 8.793) = 7.372, P=0.0176<br>multiple comparisons:<br>before vs on, P =0.0022<br>before vs after, P = 0.2357<br>on vs after, P =0.3462

Fig.6J	YFP:<br>Off:715±29.75<br>On:719.1±24.18<br>Off:726.9±29.88<br>ChR2:<br>Off:692.1±17.19<br>On:622.4±25.42<br>Off:652.1±26.08	YFP : 5mice<br>ChR2 : 7mice	Both YFP and ChR2:one-way repeated-measures ANOVA factor:<br>treatment(before,on,after)<br>Turkey's multiple comparisons test	YFP:treatment:F (1.112, 4.447) = 0.9374, P=0.3948<br>multiple comparisons:<br>before vs on, P =0.9364<br>before vs after, P = 0.0798<br>on vs after, P =0.6905<br>ChR2:treatment:F (1.214, 7.283) = 10.30, P=0.0119<br>multiple comparisons:<br>before vs on, P =0.0314<br>before vs after, P = 0.0844<br>on vs after, P =0.0340
Fig.6L	YFP:<br>Off:76.52±7.62<br>On:87.08±3.57<br>Off:87.56±4.26<br>GtACR1:Off:76.71±4.05<br>On:35.05±2.14<br>Off:69.04±7.31	YFP : 5mice<br>GtACR1: 5mice	Both YFP and GtACR1:one-way repeated-measures ANOVA factor:<br>treatment(before,on,after)<br>Turkey's multiple comparisons test	YFP:treatment:F (1.730, 6.922) = 1.134,P=0.3652<br>multiple comparisons:<br>before vs on, P =0.4205<br>before vs after, P = 0.5487<br>on vs after, P =0.9976<br>GtACR1:treatment:F (1.683, 6.734) = 24.52, P=0.0010<br>multiple comparisons:<br>before vs on, P =0.0029<br>before vs after, P = 0.6040<br>on vs after, P =0.0111
Fig.6M	YFP:<br>CS:85.33±3.75<br>CS+light:88.42±2.3<br>GtACR1:CS:85.62±3.75<br>CS+light:60.59±4.94	YFP : 5 mice<br>GtACR1: : 10 mice	YFP:two-tailed paired t test<br>GtACR1:two-tailed paired t test	YFP: P=0.1692,t=1.675, df=4<br>GtACR1:P <0.0001,t=7.591, df=9
Fig.S2C	mCherry:1.74±0.27<br>hM3Dq:32.9±3.34	mCherry:10 mice<br>hM3Dq:7 mice	two-tailed unpaired t test	P<0.0001,t=11.23, df=15
Fig.S2D	mCherry:23.85±1.27<br>hM3Dq:3.89±1.06	mCherry:10 mice<br>hM3Dq: 7 mice	two-tailed unpaired t test	P<0.0001,t=11.33, df=15
Fig.S2E	mCherry:23.5±2.64<br>hM3Dq:4.14±1.32	mCherry:10 mice<br>hM3Dq:7 mice	two-tailed unpaired t test	P<0.0001,t=5.753, df=15
Fig.S3C	Vgat:372.8±13.97<br>Vglut2:140±19.21	4 mice	two-tailed paired t test	P=0.0016,t=11.04, df=3
Fig.S3D	mCherry:<br>Light off:0.71±0.5<br>Light on:1.81±0.31<br>ChR2:<br>Light off:1.11±0.56<br>Light on:1.19±0.44	mCherry:7 mice<br>ChR2:6 mice	mCherry:two-tailed paired t test;<br>ChR2:two-tailed paired t test	P=0.1704,t=1.557, df=6;<br>P=0.8835,t=0.1542, df=5
Fig.S4B	YFP:<br>Off:8.99±1<br>On:6.35±1.72<br>Off:5.11±1.07<br>GtACR1:<br>Off:9.71±1.21<br>On:6.56±0.51<br>Off:6.03±0.84	YFP : 6mice<br>GtACR1: 9mice	two-way repeated-measures ANOVA factor one: virus (YFP, GtACR1)<br>factor two: laser(off, on,off)<br>Bonferroni's multiple comparisons test	virus: F (1, 13) = 0.2546,P=0.6223<br>interaction: F (2, 26) = 0.1051,P=0.9006<br>multiple comparisons:<br>off:YFP vs. off:GtACR1 , P>0.9999<br>on:YFP vs. on:GtACR1 P >0.9999<br>off:YFP vs. off:GtACR1 , P>0.9999
Fig.S4C	YFP:<br>Off:0.28±0.02<br>On:0.24±0.03<br>Off:0.27±0.02<br>GtACR1:Off:0.28±0.02<br>On:0.22±0.02<br>Off:0.24±0.03	YFP : 6mice<br>GtACR1: 9mice	two-way repeated-measures ANOVA factor one: virus (YFP, GtACR1)<br>factor two: laser(off, on,off)<br>Bonferroni's multiple comparisons test	virus: F (1, 13) = 0.2074,P=0.6563<br>interaction: F (2, 26) = 0.3318,P=0.7206<br>multiple comparisons:<br>off:YFP vs. off:GtACR1 , P>0.9999<br>on:YFP vs. on:GtACR1 P >0.9999<br>off:YFP vs. off:GtACR1 , P>0.9999
Fig.S4D	YFP:<br>Off:0.05±0.005<br>On:0.035±0.01<br>Off:0.028±0.006<br>GtACR1:<br>Off:0.054±0.007<br>On:0.036±0.003<br>Off:0.034±0.005	YFP : 6mice<br>GtACR1: 9mice	two-way repeated-measures ANOVA factor one: virus (YFP, GtACR1)<br>factor two: laser(off, on,off)<br>Bonferroni's multiple comparisons test	virus: F (1, 13) = 0.2581,P=0.6200<br>interaction: F (2, 26) = 0.1191,P=0.8882<br>multiple comparisons:<br>off:YFP vs. off:GtACR1 , P>0.9999<br>on:YFP vs. on:GtACR1 P >0.9999<br>off:YFP vs. off:GtACR1 , P>0.9999

Fig.S4F	YFP:<br>Off:29.5±9.24<br>On:37.43±10.54<br>GtACR1:<br>Off:32.31±9.26<br>On:27.25±7.18	YFP : 7mice<br>GtACR1: 8mice	two-way repeated-measures ANOVA factor one: virus (YFP, GtACR1)<br>factor two: laser(off, on)<br>Bonferroni's multiple comparisons test	virus: F (1, 13) = 0.1322 P=0.7220<br>laser: F (1, 13) = 0.03334 P=0.8579<br>interaction: F (1, 13) = 0.6849 P=0.4228<br>multiple comparisons:<br>off:YFP vs. off:GtACR1 , P=0.9704<br>on:YFP vs. on:GtACR1, P =0.6799<br>YFP: on vs. YFP: on, P > 0.9999<br>GtACR1: off vs. GtACR1: on, P >0.9999
Fig.S4G	YFP:<br>Off:45.72±6.74<br>On:42.21±6.57<br>GtACR1:Off:50.52±8.67<br>On:53.35±7	YFP : 7mice<br>GtACR1: 8mice	two-way repeated-measures ANOVA factor one: virus (YFP, GtACR2)<br>factor two: laser(off, on)<br>Bonferroni's multiple comparisons test	virus: F (1, 13) = 0.7108 P=0.4144<br>laser: F (1, 13) = 0.005402 P=0.9425<br>interaction:F (1, 13) = 0.4876 P=0.4973<br>multiple comparisons:<br>off:YFP vs. off:GtACR1 , P=0.8779<br>on:YFP vs. on:GtACR1, P =0.5071<br>YFP: on vs. YFP: on, P > 0.9999<br>GtACR1: off vs. GtACR1: on, P >0.9999
Fig.S5C	More water:0.31±0.22<br>Less water:76.27±5.63	ChR2:8 mice	two-tailed paired t test	P<0.0001,t=13.57, df=7
Fig.S6B	Off:3.45±2.28<br>On:32.01±7.26	6 mice	two-tailed paired t test	p=0.0076, t=4.312, df=5
Fig.S6C	Off:0.93±0.61<br>On:4.47±3.04	5 mice	two-tailed paired t test	p=0.3447,t=1.071, df=4
